# General Pyrolysis for High-Loading Transition Metal Single Atoms on 2D-Nitro-Oxygeneous Carbon as Efficient ORR Electrocatalysts

**DOI:** 10.1021/acsami.3c18548

**Published:** 2024-02-17

**Authors:** Teera Butburee, Jitprabhat Ponchai, Pongtanawat Khemthong, Poobodin Mano, Pongkarn Chakthranont, Saran Youngjan, Jakkapop Phanthasri, Supawadee Namuangruk, Kajornsak Faungnawakij, Xingya Wang, Yu Chen, Lijuan Zhang

**Affiliations:** † National Science and Technology Development Agency, National Nanotechnology Center, 111 Thailand Science Park, Pathum Thani 12120, Thailand; ‡ Shanghai Synchrotron Radiation Facility, Shanghai Advanced Research Institute, Chinese Academy of Sciences (CAS), No. 239, Zhangheng Rd., New Pudong District, Shanghai 201204, P.R. China

**Keywords:** single-atom catalysts, 2D nanomaterials, coordinative environment, oxygen reduction reaction, electrocatalysts

## Abstract

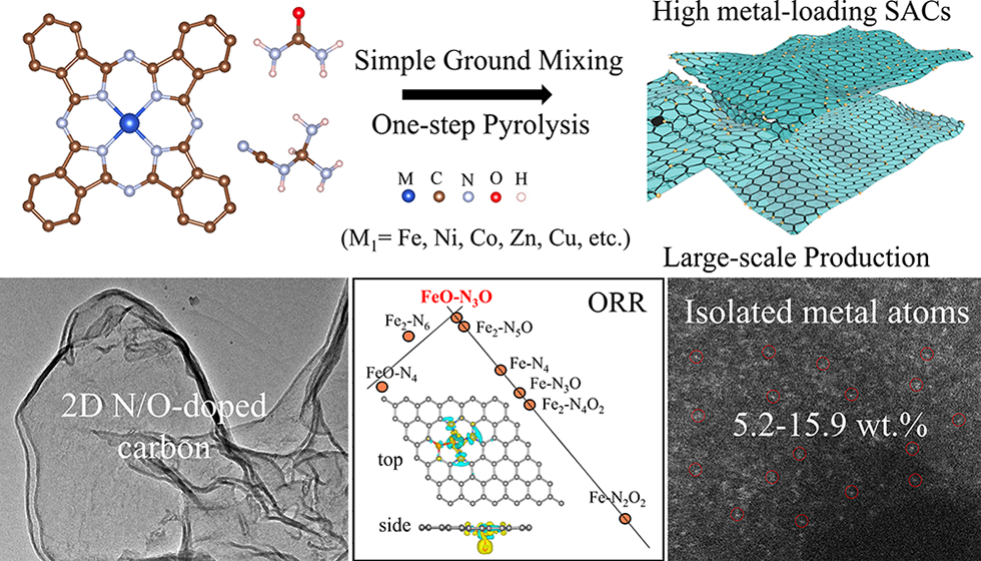

Single-atom catalysts (SACs) possess the potential to involve the merits of both homogeneous and heterogeneous catalysts altogether and thus have gained considerable attention. However, the large-scale synthesis of SACs with rich isolate-metal sites by simple and low-cost strategies has remained challenging. In this work, we report a facile one-step pyrolysis that automatically produces SACs with high metal loading (5.2–15.9 wt %) supported on two-dimensional nitro-oxygenated carbon (M_1_-2D-NOC) without using any solvents and sacrificial templates. The method is also generic to various transition metals and can be scaled up to several grams based on the capacity of the containers and furnaces. The high density of active sites with N/O coordination geometry endows them with impressive catalytic activities and stability, as demonstrated in the oxygen reduction reaction (ORR). For example, Fe_1_-2D-NOC exhibits an onset potential of 0.985 V vs RHE, a half-wave potential of 0.826 V, and a Tafel slope of −40.860 mV/dec. Combining the theoretical and experimental studies, the high ORR activity could be attributed its unique FeO-N_3_O structure, which facilitates effective charge transfer between the surface and the intermediates along the reaction, and uniform dispersion of this active site on thin 2D nanocarbon supports that maximize the exposure to the reactants.

## 1 Introduction

Distinctive advantages of homogeneous (HOM) and heterogeneous (HET) catalysts make them be the two parallel frontiers of catalysis, but they also have their own drawbacks. HOMs have outstanding benefits arising from their ultimate efficiency of atomic utilization and well-defined and uniform active sites, which lead to high chemical selectivity and reactivity. However, separation difficulties, relatively low stability, and recyclability are their important disadvantages. In contrast, HETs are appealing due to their separability, excellent recyclability, and durability. On the other hand, their nonuniform active sites and low atomic utilization efficiency usually lead to lower chemical selectivity and reactivity compared to HOMs, as only limited numbers of active sites on the catalysts’ surface accessible to the reactants can play roles in reactions. There has been an idea to incorporate the merits of HOMs and HETs altogether by fixing the homogeneous catalysts such as organometallic complexes on solid substrates,^1−4^ but this concept has not been widely applicable in industry yet, in spite of making great research efforts. This could possibly be because of the low endurance and complexity of the synthesis. Recently, single-atom catalysts (SACs) have increasingly gained enormous attention as a promising candidate to bridge this gap.^5^ When metals acting as active sites are atomically bonded on substrates and used as heterogeneous catalysts, nearly 100% atomic utilization efficiency similar to HOMs is expected^6^ while the catalysts can be easily separated from the reaction like HETs. Remarkably high catalytic activity and selectivity of SACs compared to micro- and nanosized catalysts have been reported in a wide variety of applications,^7−16^ making the research on SACs one of the hottest topics in recent years.

However, several significant challenges confine SACs to laboratory scales. First, SACs are usually derived from complicated synthesis or multistep approaches, such as MOF-derived synthesis,^17^ defect creation,^18^ and photodeposition.^19^ For example, in the case of COF/MOF-derived synthesis, which is the most employed technique to synthesize SACs on carbon supports,^20−22^ MOFs that contain target and sacrificial metals bonded with N-containing ligands are synthesized first. Then, the as-prepared MOFs are carbonized, followed by acid treatment to remove the sacrificial metals. For instance, Chen et al. used the zirconium-based MOF, UiO-66-NH_2_, to encapsulate tungsten (W) atoms.^23^ Then, UiO-66-NH_2_ with W atoms in its skeleton was carbonized at 950 °C, followed by removal of the sacrificial zirconium in hydrofluoric acid to achieve the target W-SAC. This conventional method employs a large quantity of solvents and multistep synthesis to achieve a few milligrams of catalysts. Second, the high-precision techniques that can control metal dispersion at the atomic level such as ion bombardment,^24^ mass selection with soft landing, and atomic-layer deposition^25^ also require expensive precursors and sophisticated instruments, which are unavailable in general laboratories. Third, most previously reported SACs usually have low metal loading content (<4 wt %),^26,27^ which could have insufficient active sites for target reactions. Due to the high surface energy of the isolated metal atoms, Oswald ripening spontaneously occurs especially when the loading content is increased,^28^ leading to difficulty in achieving SACs with high metal loading. Moreover, these complexities and limitations make their large-scale or industrial applications challenging.

In this work, we report a one-step pyrolysis technique to synthesize SACs with high metal loading content (>5.2–15.9 wt %) supported on two-dimensional (2D) nitro-oxygenated carbon (assigned as M_1_-2D-NOC thereafter). The synthesis can be accomplished by calcination of the blends of metal-phthalocyanine (M-Phc), dicyandiamide (Dicy), and urea in a muffle or tube furnace, which is affordable in general laboratories and can be up-scaled to several grams per batch, depending on the container size. Herein, we emphasize the low-cost transition metals such as Fe, Ni, Co, Zn, and Cu. The synthesis principle could also be applicable to other metals. The isolated metal characteristics and their oxidation states as well as coordination geometries were insightfully investigated by aberration-corrected scanning transmission electron microscopy (ABF-STEM), high-angle annular dark-field STEM (HAADF), extended X-ray absorption fine structure (EXAFS), X-ray absorption near edge structure (XANES), and X-ray photoelectron spectroscopy (XPS), while their physicochemical properties were studied by various advanced characterizations such as transmission electron microscopy (TEM) equipped energy-dispersive spectroscopy (EDS), X-ray powder diffraction (PXRD), Fourier transform infrared spectroscopy (FTIR), Raman spectroscopy, and Brunauer–Emmett–Teller (BET) surface area analyzer. The catalytic performance of the catalysts was assessed toward the oxygen reduction reaction (ORR) using a rotating ring-disk electrode. Fe_1_-2D-NOC demonstrated the highest catalytic performance, with an onset potential and half-wave potential of 0.985 and 0.826 V vs RHE, respectively, and a nearly perfect 4-e^–^ transfer pathway. The activity of Fe_1_-2D-NOC is followed by Cu_1_-2D-NOC, Ni_1_-2D-NOC, Co_1_-2D-NOC, and Zn_1_-2D-NOC, respectively. Their catalytic behaviors were also theoretically studied by density functional theory (DFT) simulation. Combined with experimental and theoretical results, it was found that the superior catalytic performance of Fe_1_-2D-NOC could be attributed to its unique FeO-N_3_O active site, which facilitates the more effective charge transfer between the surface and the intermediates along the key steps of reaction.

## 2 Results and Discussion

**Figure 1:**
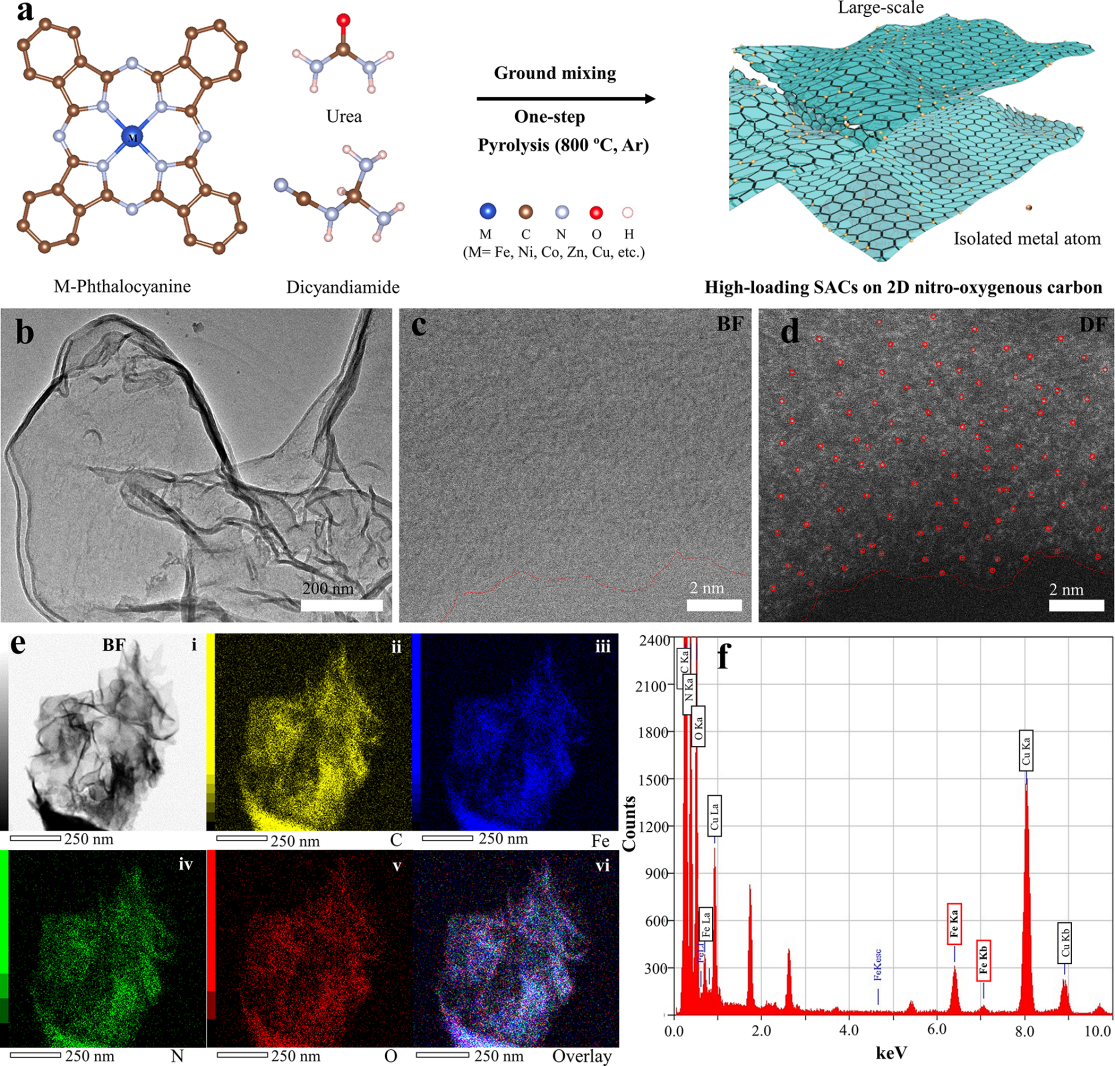
(a) Schematic figure illustrating the synthesis approach for M_1_-2D-NOCs. (b) TEM image showing the morphology of the resultant M_1_-2D-NOC products (Fe_1_-2D-NOC in this case). (c, d) Aberration-corrected ABF-STEM and aberration-corrected HAADF-STEM images of Fe_1_-2D-NOC, respectively (c and d were taken at an identical area). e(i) Bright-field STEM, and e(ii–vi) EDS mapping of Fe_1_-2D-NOC, consisting of C (ii), Fe (iii), N (iv), and O (v), and the overlay map (vi). (f) EDS spectrum of the Fe_1_-2D-NOC particle (Cu signals are from the Cu TEM grid).

M_1_-2D-NOCs were synthesized by pyrolysis of an M-Phc, Dicy, and urea mixture (details in Section 4). N and O are reported as coordinating atoms that can tenaciously tie with single metal atoms, preventing them from aggregation.^27,29,30^ Therefore, Dicy and urea were purposively chosen as precursors because they are small molecules containing high N and O contents. At the same time, M-Phc is a macrocyclic molecule, in which a single metal is bonded with well-ordered N atoms, making it a readily single-metal-site pocket. The typical synthesis procedure is summarized, as shown in Figure 1a. After these molecules with a suitable ratio (details in Section 4) were simply ground-mixed, they underwent polymerization and carbonization simultaneously during one-step pyrolysis. The resultant products autonomously formed 2D carbonaceous nanostructures, as shown in the TEM image (Figure 1b). The samples were further stirred in 37% HCl and washed with water to ensure full exfoliation of the 2D nanosheets and automatically dissolve possible metal residues. There are no metal nanoparticles on the 2D nanosheets when observed by either low-magnification TEM (Figure 1b) or high-resolution TEM image (Figure 1c). On the other hand, EDS mapping shows that the metal is densely distributed throughout the whole nanosheets, as shown in Figure 1e-iii. Similarly, the EDS spectrum (Figure 1f) also shows a high-intensity Fe signal. We use Fe_1_-2D-NOC to discuss in this main text as the example showcase, while EDS mapping of the other M_1_-2D-NOCs can also be found in Supporting Information Figure S1. High-angle annular dark-field STEM (HAADF-STEM) image (Figure 1d), which was taken at an area identical to that of the bright-field image (Figure 1c), reveals the atomically dispersed Fe atoms (bright spots, which are selectively highlighted in the red circles) with high density without metal clusters observed. The larger HAADF-STEM images showing clearer dispersion of the high-density single atoms can be found in Supporting Information S2. The microscopic characterizations agree well with the XRD results, in which only characteristic peaks of graphitic carbon are observed without those of metals (Supporting Information Figure S3). The metal contents in M_1_-2D-NOCs analyzed by inductively coupled plasma atomic emission spectroscopy (ICP-AES) were 5.2–7.5 wt % (Table 1), which are among the highest-loading SACs that have been recently reported.^26,31^ The content of N, the element that plays key roles on anchoring isolated metal atoms,^30^ was also quantified by the CHONS technique. As summarized in Table 1, all samples have a similar N content (∼22–25 wt %). The metal loading content appears to be varied, with respect to the N content. For example, Ni_1_-2D-NOC that contains the highest N content (25.22 wt %) also has the highest metal loading content (7.57 wt %) among the M_1_-2D-NOCs. Note that the concentration of some metals (such as Cu and Zn) in the form of isolated atoms could be further increased to >15 wt % using our synthesis method,^32^ but in this work, we keep the concentration to ∼5–8 wt % where all types of metals remain well atomically dispersed and synthesized using a similar recipe (see Section 4) for the fair comparison of their catalytic performances.

**Table 1:** Summary of Physicochemical Properties and ORR Parameters of Various M_1_-2D-NOCs, Namely, Double-Layer Capacitance (*C*_dl_), BET Surface Area, Limiting Current Density at 0.2 V vs RHE, Onset Potential, Half-Wave Potential, Electron Transferred Numbers (*n*) at 0.2 V vs RHE, and Mass Activity at 0.85 V vs RHE

	**catalysts**				
**parameters**	**Fe_1_-2D-NOC**	**Cu_1_-2D-NOC**	**Ni_1_-2D-NOC**	**Co_1_-2D-NOC**	**Zn_1_-2D-NOC**
*J* at 0.2 V (mA/cm^2^) at 1600 rpm	–5.22	–5.08	–4.06	–2.56	–1.32
*E*_onset_ (V)	0.985	0.893	0.845	0.877	0.809
*E*_1/2_ (V)	0.826	0.774	0.685	0.665	0.694
Tafel slope	–40.86	–50.75	–78.68	–75.21	–135.50
mass activity at 0.85 V (A/mg)	2.05	0.142	0.063	0.026	0.134
*n* at 0.2 V	4.00	3.95	3.60	2.22	2.05
metal loading content (wt %, ICP-AES)	5.20	5.66	7.57	5.92	5.55
*N* content (wt %, CHONS)	21.95	23.01	25.22	24.42	23.06
*C*_dl_ (mF/cm^2^)	19.59	21.33	6.07	0.34	2.57
BET surface area (m^2^/g)	183.8	181.3	261.8	212.3	197.7

**Figure 2:**
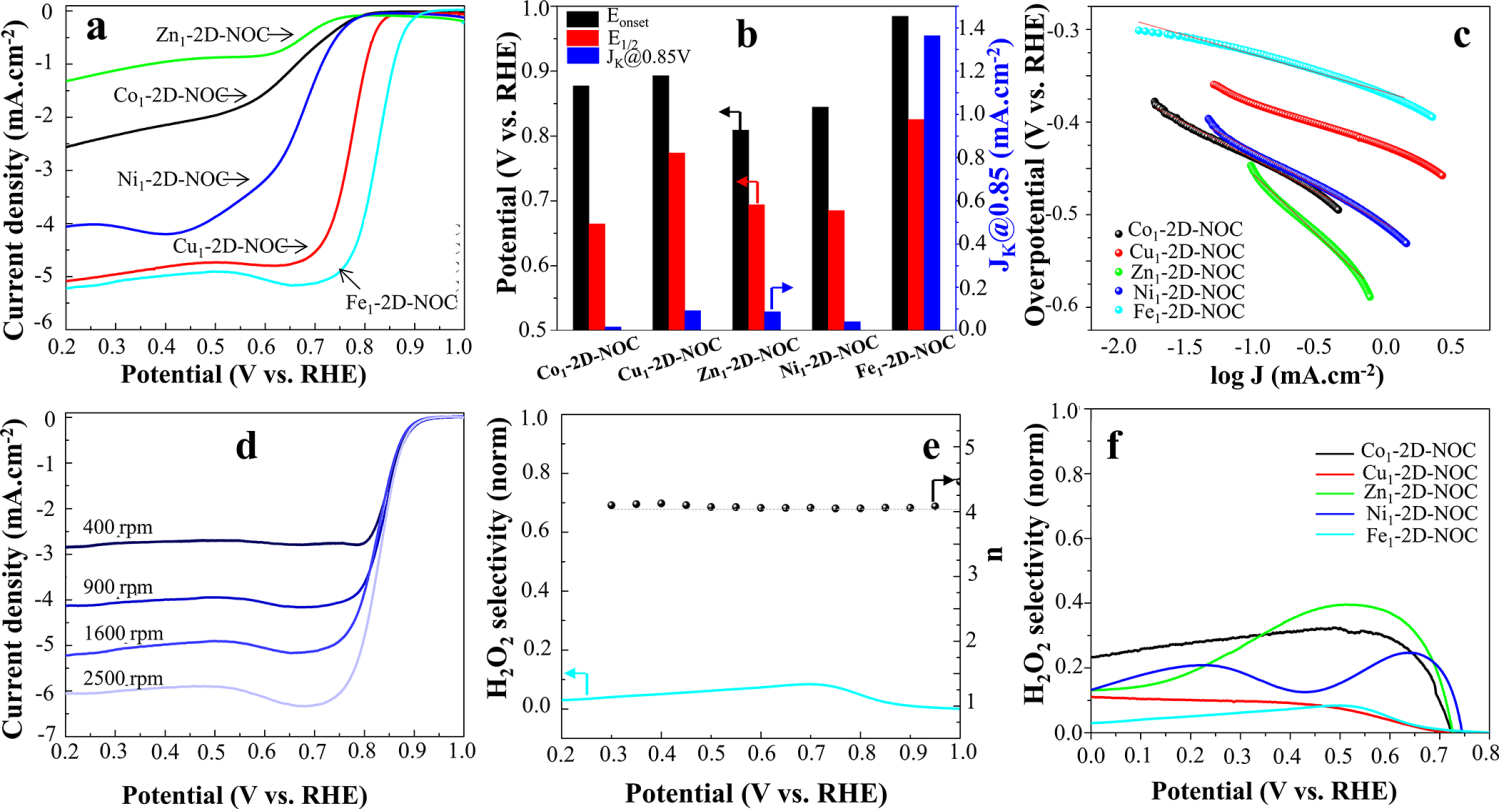
(a) ORR polarization plots of M_1_-2D-NOCs, where M = Co, Cu, Zn, Ni, and Fe. (b) The performance parameters, namely, onset potential (*E*_onset_), half-wave potential (*E*_1/2_), and kinetic current density (*J*_K_) of various M_1_-2D-NOC catalysts. (c) Tafel plots of various M_1_-2D-NOC catalysts. (d) LSV curves of Fe_1_-2D-NOC at different rotation rates. (e) ORR pathway selectivity and electron numbers (*n*) at various potentials, using Fe_1_-2D-NOC catalysts in O_2_-saturated 0.1 M KOH. (f) H_2_O_2_ selectivity using various M_1_-2D-NOC catalysts.

We performed activity screening to preliminarily evaluate the catalytic performances of the as-prepared catalysts with different transition metal active sites (M_1_-2D-NOCs, where M = Co, Cu, Zn, Ni, and Fe) toward the ORR in 0.1 M KOH electrolyte. The results showed that Fe_1_-2D-NOC exhibited the most promising ORR activity, followed by Cu_1_-2D-NOC, Ni_1_-2D-NOC, Co_1_-2D-NOC, and Zn_1_-2D-NOC, respectively. As shown in the linear sweep voltammetry curves (Figure 2a), Fe_1_-2D-NOC has the most positive onset potential (*E*_onset_) of 0.985 V vs the reversible hydrogen electrode (RHE) while those of Cu_1_-2D-NOC, Ni_1_-2D-NOC, Co_1_-2D-NOC, and Zn_1_-2D-NOC are 0.893, 0.845, 0.877, and 0.809, respectively. Clearly, Fe_1_-2D-NOC delivered significantly higher kinetic current density (*J*_K_) of −1.36 mA/cm^2^ at 0.85 V vs RHE and half-wave potential (*E*_1/2_) of 0.826 V vs RHE compared to those of the other M_1_-2D-NOCs, as summarized in Figure 2b as well as Table 1. In addition, the smallest Tafel slope of −40.86 mV/dec is observed for Fe_1_-2D-NOC (Figure 2c and Table 1), suggesting that it has the fastest ORR kinetics.^33−35^ The steady-state linear sweep voltammogram (LSV) of Fe_1_-2D-NOC on rotating ring-disk electrodes (RRDE) at varying speeds and the corresponding calculated electron transfer (*n*) are respectively shown in Figure 2d,e (those of the other M_1_-2D-NOC catalysts can also be found in Supporting Information S4). As shown in Figure 2e, a nearly perfect 4-e^–^ ORR with negligible hydrogen peroxide production was observed throughout the potential range of 0.2–0.9 V vs RHE while the other M_1_-2D-NOCs generate significantly higher H_2_O_2_ values, as shown in Figure 2f. These parameters indicate the superior performance of Fe_1_-2D-NOC, on par with the recently reported state-of-the-art ORR catalysts (Supporting Information S5). Basically, Fe_1_-2D-NOC still has slightly inferior *E*_onset_ and *E*_1/2_ than that of the benchmark Pt/C catalyst, but it has much higher stability and methanol tolerance, as separately discussed in Supporting Information S6. Surprisingly, the ORR performance seems to be independent of either surface area or metal loading contents in this study (a comparison can be found in Table 1). For example, Fe_1_-2D-NOC has the smallest metal content and almost the lowest BET surface, while the pore size distribution of all catalysts is similar (see Supporting Information S7), but it shows the highest ORR performance. In addition, the order of ORR performance does not follow the order of Fe > Co > Cu > Ni, which is the general trend for the conventional M_1_-N_4_ active sites.^36,37^ These results suggest that the additional O atoms could change the nature of active sites and the resulting properties of these materials, which is in accordance with the previous report.^38^

**Figure 3:**
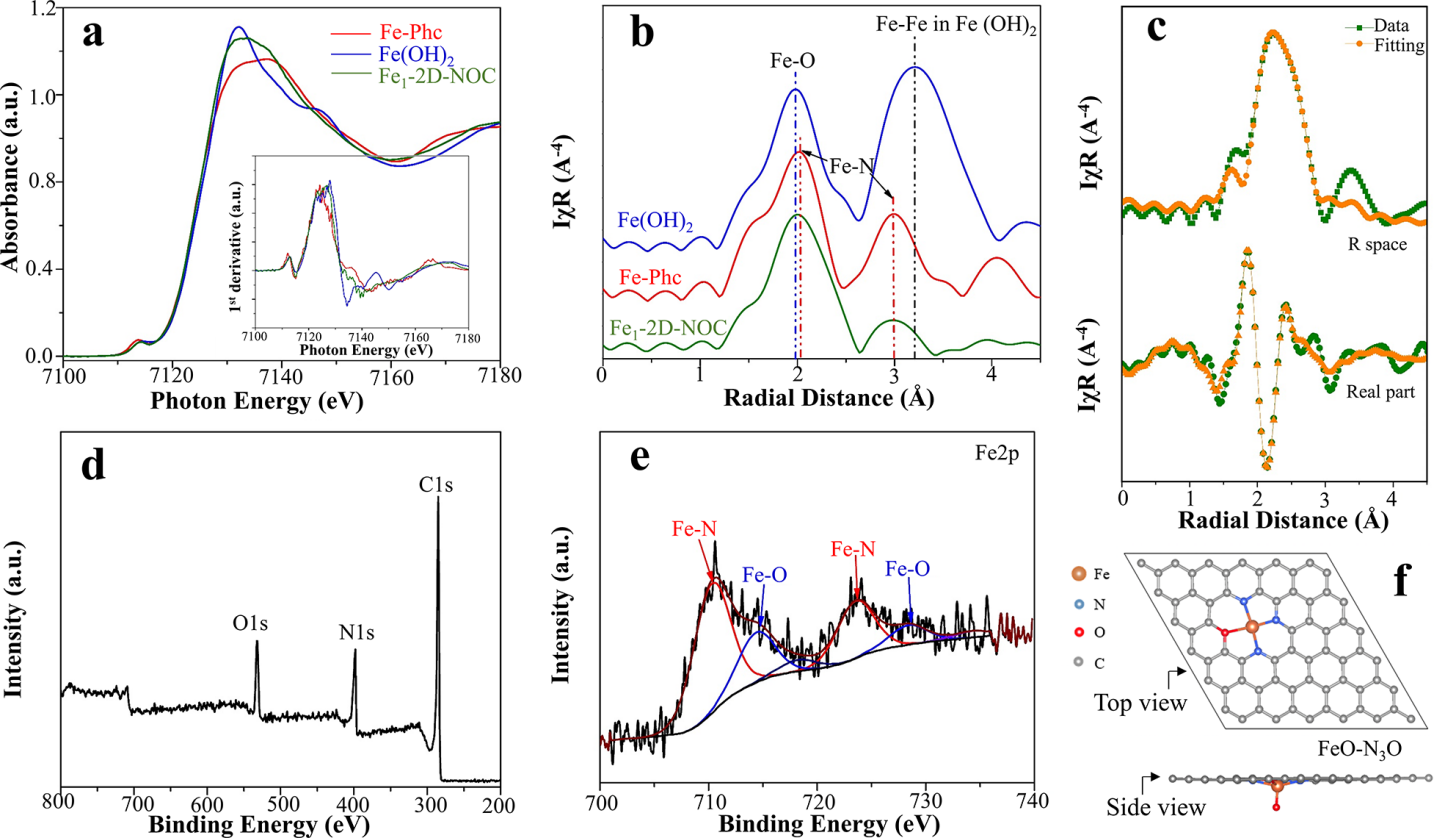
(a) Fe K-edge XANES spectra compared with standard materials (the inset is the first derivative). (b) Fourier-transformed R space. (c) Corresponding Fe K-edge EXAFS fitting curves (the upper lines represent the R space; the lower lines are the real part). (d) XPS survey scan with details of the element contents. (e) XPS Fe 2p of Fe_1_-2D-NOC with Fe–N and Fe–O fittings. (f) Proposed structure of the major active site in Fe_1_-2D-NOCs.

Therefore, we carefully investigated the nature of active sites by various advanced X-ray characterizations such as XANES, EXAFS, XRD, and XPS techniques to gain insight into the coordinative environment and oxidation number of the metal active sites. Particular attention is therefore paid to Fe_1_-2D-NOC, the most active ORR catalyst among the studied samples. The XANES spectrum of Fe_1_-2D-NOC compared to that of the other standard materials, namely, Fe-Phc and Fe(OH)_2_, is shown in Figure 3a. It is noticeable that the characteristics of the XANES spectrum of Fe_1_-2D-NOC are significantly changed compared to those of the Fe-Phc precursor, especially the white line peak. This implies that the coordination and geometry of the Fe atoms are altered after the thermal treatment process. The edge of Fe_1_-2D-NOC is located at a similar position to Fe-Phc and Fe(OH)_2_ standards (inset Figure 3a), indicating that the valence of the major Fe atoms in Fe_1_-2D-NOC could be ∼Fe^2+^. There is no characteristic peak of the metallic Fe–Fe bond (2.2 Å) observed in the spectrum space of Fe_1_-2D-NOC (Figure 3b), which further verifies the isolated nature of the metal atoms, in agreement with the TEM and XRD results. The Fourier transformed EXAFS spectrum (Figure 3c) shows the dominant peaks of Fe–O and Fe–N with coordination numbers of 1.95 (bond length of 1.96 Å) and 2.92 (bond length of 2.12 Å) with nearly perfect fitting parameters (see Supporting Information S8), respectively. XANES and Fourier-transformed EXAFS spectra of the other M_1_-2D-NOCs are also available in Supporting Information S9. The XPS survey spectrum in Figure 3d confirms the coexistence of C, N, O, and Fe. Detailed deconvolution of the Fe 2p spectrum is shown in Figure 3e. The XPS spectrum of Fe 2p shows both the Fe 2p 1/2 and 3/2 doublets that represent Fe–N and Fe–O species, confirming the bonding of Fe with N and O atoms. We found that a suitable content of the O dopant in the N-doped graphitic carbon structure can significantly enhance the catalytic performance compared to the conventional pure N-coordinated SACs (such as FeN_4_),^39−41^ which will be discussed in the next section.

**Figure 4:**
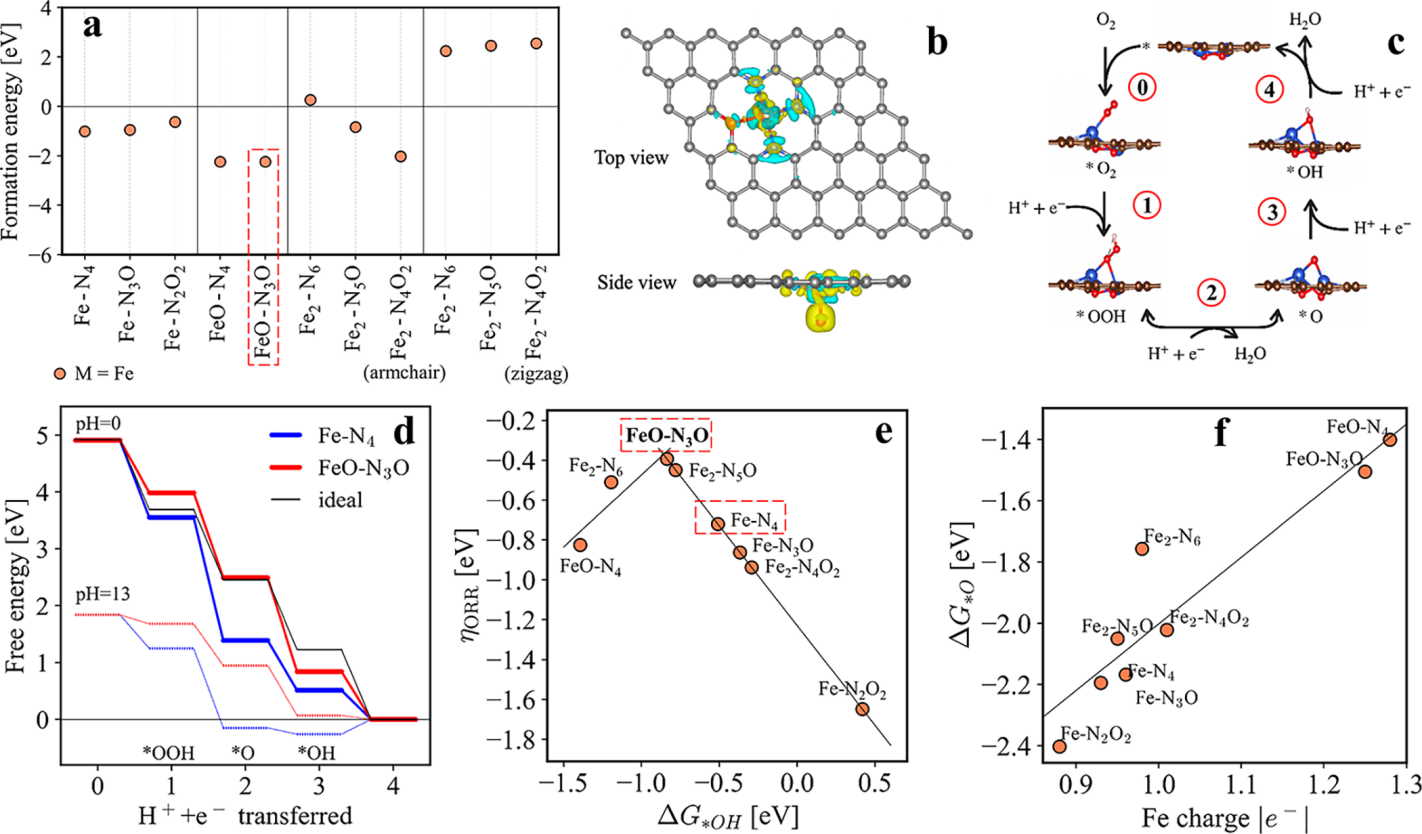
(a) The formation energies of 11 of the most possible active sites in Fe_1_-2D-NOC modeled in this study. (b) Charge density difference plot around the FeO-N_3_O active site (yellow = higher e^–^ density, cyan = lower e^–^ density). (c) Schematic diagram showing the five steps of the four-electron ORR pathway, which were used for calculating the activation energies of the catalysts. (d) The calculated free energies of the four-electron ORR pathways using the FeO-N_3_O active site (red) at pH = 0 and pH = 13 (dashed line) compared to the ideal (black) and the traditional Fe–N_4_ active site. (e) Volcano plot toward the ORR potential of various Fe active sites, using ΔG_*OH_ as a descriptor. (f) The correlation between reaction descriptor ΔG_*OH_ and Fe charge.

To further verify the experimental results, the structures of the 11 most possible active sites in Fe_1_-2D-NOC were simulated based on density function theory (DFT) (Supporting Information S10). It was found that the structure of FeO-N_3_O shows the negative formation energy of −2.0 eV (Figure 4b), which is one of the most stable forms among the 11 models. This confirms the thermodynamic stability of the structure and suggests that those structures with ligands could be easily formed, which is consistent with the experimental results. Combining theoretical and experimental results (elemental analysis, XANES, EXAFS, and XPS), we therefore proposed that FeO-N_3_O (Figure 3f) could be the major active site in the Fe_1_-2D-NOC catalyst. This structure is beneficial not only in terms of stability but also in terms of enhancing ORR performance. Figure 4c shows the energy diagrams for the four-electron ORR pathway, which include O_2_ adsorption on the catalyst’s surface (denoted as *O_2_, step 0), and the following by the first, second, third, and fourth protonation to form *OOH (step 1), *O (step 2), and *OH (step 3) as descriptors and desorption to form H_2_O (step 4).^42^ Considering the energy diagrams, the FeO-N_3_O model shows superior performance with a low ORR overpotential (η_ORR_) of −0.39 V. Only this active site shows the downhill activation energies for all reaction steps (Figure 4d and Supporting Information S11), especially in the alkaline condition that was practically used in our testing system. This evidence suggests that these active sites facilitate more efficient ORR. To explain the general trend of the activity, the free energy change of *OH intermediate (ΔG_*OH_) was chosen as a descriptor for the overpotential.^43−45^ The nearly perfect volcano plot can be obtained where FeO-N_3_O is located on the top of the volcano, confirming that ΔG_*OH_ is a good descriptor for the reaction (Figure 4e). Interestingly, a greater number of O-replacement to the in-plane N-coordination would increase the overpotential and lower the ORR activity, as seen from the trend going from Fe–N_4_, N_3_O–Fe to the lowest activity of ORR = −1.65 eV for N_2_O_2_–Fe. To rationalize the general trend of the activity, the charge of the Fe active center was evaluated (see Supporting Information S12). It should be noted that instead of only discussing *OOH and *OH intermediates, *O should be also taken into account, as it essentially affects both *OOH and *OH binding. As shown in Figure 4f, the linear relationship was found between the Fe atomic charge and the free energy change at the middle step ΔG_*O_ of the reaction pathway. Taking Fe–N_4_ and N_6_–Fe_2_ as references (∼0.96|e^–^|), having more in-plane O-coordination in the support systematically decreases the positive charge of the metal, which weakens the Coulomb interaction between negatively charged O-bound species and the active site. In contrast, the presence of the ligand directly on the Fe active site results in the increase of the metal charge, which plays a significant role in enhancing the binding of the intermediate. To gain a deeper insight into the role of ligands as charge controllers, a charge density plot was calculated. As seen from Figure 4b, charge accumulation (yellow) around the O-ligand and charge depletion (blue) around the Fe atom indicate significant charge transfer from the Fe atom to the O-ligand, which agrees with the picture of the ligand as a charge regulator to retrieve electrons from the metal. This fact clearly indicates the importance of the charge regulation of the active site as the key strategy in modulating the binding of the optimized O-bound species.

Beyond the conventional M_1_-N_4_ moieties, which are usually proposed as the active sites of SACs supported on nitrogen-doped carbon (MNC) supports,^40,46^ there is a handful of reports showing that doping with additional elements (such as P, O, and S) can further enhance the electrochemical performance by involving several merits to SACs.^47−49^ First, doping with negative charge heteroatoms can alter the electron density around the active metal centers, facilitating OH release, which is generally the rate-determining step of ORR for most catalysts. For example, Chen et al.^50^ reported that the OH protonation is endothermic when Fe-SACs supported on MNC with the Fe_1_–N_4_ active site are used as a catalyst while this step becomes exothermic when Fe-SACs supported on P-doped and P/S-doped NMC with Fe_1_–N_4_P_2_ and Fe_1_–N_4_P_2_S_2_ are used. Second, the heteroatom dopants could create axial guest groups and asymmetric coordination around the active site, which are beneficial for O_2_ binding and O–O breaking.^51^ For instance, Wang and co-workers revealed that the additional axial OH ligand atop the planar Fe–N_4_ can weaken the adsorption of the ORR intermediates by down-shifting the d-band centers, which consequently leads to the apparent positive shift of *E*_onset_ and improves the overall ORR performance.^52^ These promotional effects are also in agreement with the studies by other groups.^53−56^ Furthermore, Yang et al.^38^ found that partial replacement of some N by O atoms in the planar Mn_1_–N_4_ can generate asymmetric coordination geometries such as Mn_1_–N_3_O, Mn_1_–N_2_O_2_, and Mn_1_–N_1_O_3_, leading to optimization of the electron density in Mn (d-orbital). This improves the intrinsic property of Mn, which is usually the less active metal for ORR than Fe and Co,^57^ to be more active than the benchmark Pt/C catalyst. Moreover, the asymmetric active sites also encourage the breakaway of the O–O bond and desorption of ORR intermediates, as verified by many studies.^47,58,59^ The unique FeO-N_3_O active site (Figure 3f) proposed in this work, which is constructed from both asymmetric Fe–N_3_O and axial OH, could employ the synergy of the aforementioned effects. DFT simulation results reveal that doping with more negative charge O atoms on the planar surface and on the axial position causes a reduction of the electron density around the Fe active metal centers (see comparison of electron density in the traditional Fe–N_4_ and FeO-N_3_O in Supporting Information S13). This results in weakening of the binding energy of *O and *OH intermediates, which is a limiting step in the Fe–N_4_ catalyst (dashed blue line in Figure 4d). For the FeO-N_3_O active site, the binding energy of *O and *OH intermediates is shifted to the ideal catalyst, leading to a promising ORR performance.

## 3 Conclusions

In conclusion, we report facile one-step pyrolysis to synthesize SACs with high metal loading on N/O-doped carbon nanosheets. The method is generic to several transition metals such as Fe, Ni, Co, Zn, and Cu. The resultant materials can be used as low-cost and efficient catalysts, as demonstrated in the ORR. For example, Fe_1_-2D-NOC shows an onset potential of 0.985 V vs RHE, a half-wave potential of 0.826 V, and a Tafel slope of −40.86 mV/dec. The unique FeO-N_3_O active site, which facilitates the adsorption, protonation, and desorption of O_2_ molecules, could contribute to the impressive ORR performance. Moreover, this high-density active site is supported on thin 2D nanosheets that maximize exposure to the reactant, leading to an impressive ORR performance. Our developed method for synthesizing SACs is simpler than the conventional methods that usually require complicated procedures, sophisticated instruments, or expensive reagents such as MOF/COF-derived synthesis, defect creation, ion bombardment, and ligand chelation. Thus, this finding will make high-quality SACs affordable, easily repeatable, and scalable in general laboratories, which could further drive SACs to practical and widespread applications.

## 4 Methods

### 4.1 Chemicals

Cobalt phthalocyanine (Co-Phc, 97%), copper phthalocyanine (Cu-Phc, 90%), Cu(NO_3_)_2_·2.5H_2_O (98%), Zn(NO_3_)_2_·6H_2_O (98%), KOH (99%), and Nafion solution (5 wt %) were purchased from Sigma-Aldrich. Zinc phthalocyanine (Zn-Phc, 96%), nickel phthalocyanine (Ni-Phc, 95%), iron phthalocyanine (Fe-Phc, 96%), and dicyandiamide (Dicy, 99.5%) were purchased from Acros Organics. Urea (ACS grade) and HCl (37%) were purchased from Carlo Erba. Co(NO_3_)_2_·6H_2_O (AR grade) and Fe(NO_3_)_3_·9H_2_O (ACS grade) were purchased from Univar. Ni(NO_3_)_2_·6H_2_O (ACS grade) was purchased from Unilab. Isopropanol (IPA, AR grade) was purchased from Fisher Chemicals. The commercial platinum–carbon catalyst (Pt/C, 46.5 wt %) was purchased from Tanaka Kikinzoku International Co., Ltd. Carbon Black (TIMCAL Super P) was purchased from XIAMEN TOB New Energy Technology Co., Ltd. No further purification is needed for all chemicals. Generally, deionized (DI) water (type 2) was used as a solvent for all experiments, except other special solvents mentioned in a specific section.

### 4.2 Synthesis of M_1_-2D-NOCs

Desirable M-Phc, urea, and Dicy with a weight ratio of 0.3:5:5 g were ground-mixed thoroughly together. Then, the mixture was placed in a quartz boat that was closed with a lid and pyrolyzed at 800 °C (ramp rate = 10 °C·min^–1^) in a tube furnace under an Ar atmosphere (flow rate = 200 sccm). Note that metal loading contents can be adjusted by tuning the ratios of M-Phc:Dicy:urea as described in detail in our previous report.^32^ Basically, urea tends to carry a higher metal loading than Dicy, as it is a smaller molecule with high N and O contents that are beneficial for anchoring isolated metal atoms. However, the resulting 2D materials seem to become more stacked when the ratio of urea is higher (see Supporting Information S14). On the other hand, Dicy appears to promote better exfoliation of the 2D structure and consequently increase the specific surface area. Therefore, the ratio between M-Phc:Dicy:urea should be optimized to achieve high metal loading and well-exfoliated 2D nanosheets. For example, 15.9 wt % Cu loading content with a suitably exfoliated 2D structure could be achieved when the weight ratio of M-Phc:Dicy:urea of 1:5:5 was used.^32^ Nevertheless, some metals are supposed to form clusters more easily than the others, depending on the nature of the metals. In the present work, we used a ratio of 0.3:5:5 g to ensure that all metal catalysts remained in an atomically dispersed form supported on well-exfoliated thin 2D-NOC nanosheets. In addition, the pyrolysis can be scaled up to several grams, depending on the size of the crucible container and furnace. After maintaining at 800 °C for 2 h, the reaction vessel was allowed to naturally cool to room temperature. Then, 0.2 g of the resulting powders was dispersed in 37% HCl, which contains the same metal ions (8 mmol) as in the form of metal chlorides. For example, in the case of Fe_1_-2D-NOC, 3.27 g (8 mmol) of Fe(NO_3_)_3_·9H_2_O was mixed with 8.01 mL of 37% HCl under mechanical stirring followed by putting 0.2 g of the as-prepared Fe_1_-2D-NOC powder into this solution. Then, the mixture was ultrasonicated (150 W) in an ice-cooled ultrasonic bath for 1.5 h. This step was applied to ensure complete removal of possible metal and organic residues and to enhance exfoliation simultaneously. After that, the mixture was filtered under a vacuum and a copious amount of DI water was applied to wash the powder. The as-prepared powders were vacuum-dried in an oven that was set at 60 °C and stored at this condition for further use.

### 4.3 Material Characterization

The morphologies of various M_1_-2D-NOCs were observed by a field-emission scanning electron microscope (SEM, Hitachi SU8230) and a TEM (JEOL 2100Plus) equipped with an EDS detector. The atomically dispersed characteristics were visualized by the JEOL ARM200F atomic-resolution scanning transmission electron microscope operating at 80 keV. Bruker 1600 W (20 mA, Cu Kα radiation with λ = 1.5418 Å) was used for XRD analysis. X-ray absorption measurements (EXAFS and XANES) were carried out at Beamline 11B of Shanghai Synchrotron Radiation Facility (SSRF), China, and Beamline BL 5.3 of the Synchrotron Light Research Institute, Thailand. XAS analysis was performed with Athena and Artemis software. Phase shift was applied for the EXAFS fitting. CIF files of Fe(OH)_2_ and Fe-Phc were used as the standard modeling. XPS was measured by Axis Ultra^DLD^ (Kratos Analytical) with monochromatic Al K_α_ irradiation at 1.4 keV. A PerkinElmer Avio 200 was used for inductively coupled plasma atomic emission spectroscopy (ICP-AES) analysis. N content in the catalysts was analyzed by a CHONS elemental analyzer (LECO CHNS628) equipped with a thermal conductivity detector (TCD) at the combustion temperature of 950 °C. N_2_ adsorption–desorption isotherms were acquired from a Quantachrome Autosorb iQ3 and used for evaluating the specific surface area by the Brunauer–Emmett–Teller (BET) method.

### 4.4 Electrode Preparation

To prepare catalyst ink, 250 μL of a 1:3 volume ratio of water and IPA mixture was prepared. Then, 30 μL of 5 wt % Nafion and 4 mg of the catalyst were respectively added to the solution under continuous magnetic stirring. The mixture was then further stirred and ultrasonically sonicated (1 h). After being well dispersed, the ink was coated on a glassy carbon plate by a spin-coating technique (catalyst loading of 0.65 mg/cm^2^). Then, the catalyst-coated substrate was vacuum-dried at 60 °C for 1 h before use. A Pt/C electrode, which was used as a reference for comparison, was also prepared by the same method. 46.5 wt % Pt/C was diluted with carbon black to achieve the similar metal concentration of that in M_1_-2D-NOCs (5 wt %) before use.

### 4.5 ORR Test

A rotating ring-disk electrode (RRDE, Ametek 636A) was used for all electrochemical measurements. In short, the ORR was performed in the standard three-electrode system consisting of Ag/AgCl, a Pt wire, and a catalyst-coated glassy carbon disk as the reference, counter, and working electrodes, respectively. 0.1 M of O_2_-saturated KOH was used as an electrolyte. Linear sweep voltammetry (LSV) plots were measured at a scan rate of 10 mVs^–1^. The ring rotating rates were ranged from 400 to 2500 round per minute (rpm), and the potentials were ranged from −1.1 to 0.1 V vs Ag/AgCl. The LSV was 100% compensated for the solution resistance at open circuit potential ranging from 10,000 to 1 Hz with an amplitude of 10 mV. (*n*) and %H_2_O_2_ represent the electron transfer number, and the hydrogen peroxide yield, and were calculated according to eqs 1 and 2, respectively.
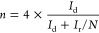
1
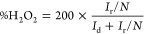
2

*I*_d_ stands for the disk current, while *I*_r_ represents the ring current. The current collection efficiency of the Pt ring (*N*) was determined to be 0.254, according to the reduction of K_3_Fe[CN]_6_.^60^

The Koutecky–Levich (K–L) equation (eq 3)^61^ was used to calculate the ORR kinetics.
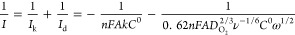
3

*I, I*_d_*,* and *I*_k_ in this equation stand for the measured current, diffusion-limiting current, and kinetic current, respectively. *n, F,* and *A* represent the electron number, Faraday constant, and geometric electrode area (cm^2^), respectively. *k*, *C*^0^, *D*_O2_, ν, and ω are the rate constant for ORR, the saturated concentration of O_2_ in KOH (0.1 M), the diffusion coefficient of O_2_, the solution kinetic viscosity, and the electrode rotation rate, respectively.

The Nernst equation (eq 4) was applied to calibrate the potentials vs Ag/AgCl to RHE.
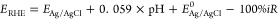
4
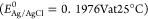


The double-layer capacitance (*C*_dl_), which signifies the electrochemically active surface area of the catalyst, was assessed by the cyclic voltammetry method under Ar-purged electrolyte between −0.2 and 0.2 V vs Ag/AgCl at varying scan rates of 5, 10, 25, 50, and 75 mV/s. The *C*_dl_ was obtained from the slope of the averaged capacitive current density plot against the scan rate.

### 4.6 Methanol Tolerance Test

Methanol tolerance test was carried out by adapting the method according to the previous report.^62^ At constant potential of 0.50 V vs RHE, 5.05 mL of methanol was spiked into 120 mL of KOH electrolyte at a desirable time (at 200 s in our study, as shown Figure S 6c), resulting in a concentration of 1 M.

### 4.7 Computational Methods

The electronic states were calculated using spin-polarized density functional theory (DFT) as implemented in Vienna Ab initial Simulation Package (VASP).^63^ The generalized gradient approximation (GGA) in the Perdew–Burke–Ernzerhof (PBE) scheme was chosen as the exchange correlation functional, while the projector augmented wave (PAW) method was chosen to describe the electron interaction. To consider the van der Waals (vdW) interaction, the DFT-D3 scheme^64^ was applied. The plane wave basis was utilized with the energy cutoff of 450 eV, while the Brillouin zone was sampled with 3 × 3 × 1 of *k*-mesh. The thresholds for the energy and force optimization were 10^–5^ eV and 0.01 eV/Å, respectively. For the SAC model, the metal embedded graphene in the 6 × 6 Å supercell with a PBE-optimized lattice constant of 14.81 Å was used. To avoid the interaction between the periodic images, 12 Å of the vacuum layer was introduced. Bader charge analysis was utilized to analyze the electronic structure using the Bader code.^65^ The thermodynamical stability was evaluated from the formation energies *E*_F_, as described by eq 5.
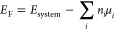
5where *E*_system_, *n_i_*, and μ_*i*_ are the total energy of the system, the number of atoms *i*, and the corresponding chemical potential, respectively. The catalytic activity was evaluated based on the computational hydrogen electrode (CHE) method developed by No̷rskov et al.,^66^ where Gibbs free energy can be expressed, as shown in eq 6.
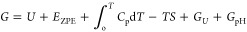
6where *U* is total energy of the complex system, *E*_ZPE_ represents the zero-point energy, and *C*_p_ and *S* are the enthalpic and entropy correction term at temperature 298 K, respectively. The applied potential correction was introduced by *G_U_* = −neU, which is considered as 0 in this study, while the pH correction is estimated from *G*_pH_ = *k*_B_*T*ln10 × pH relationship. The mechanism for ORR was considered in four electron transfer steps as described below.
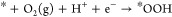

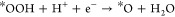

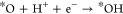

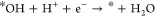
where * denotes the Fe active site. Using the equilibrium potential 1.23 eV from the experiments, the free energy change in each step can be estimated using the equations below.
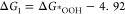

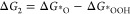

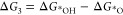

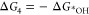
where the overpotentials are described using eq 7
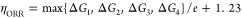
7

## Supporting Information Available

EDS mappings of various M_1_-2D-NOCs; high-resolution HAADF STEM images showing the metal population in the sample; XRD of various M_1_-2D-NOCs; rotating speed-dependent LSV of various catalysts; ORR performance of different types of metal supported on N-doped carbon; stability and methanol tolerance tests; BET surface area and pore size distribution; EXAFS fitting parameters; XANES and the first derivative of the other M_1_-2D-NOCs; simulated structures of possible active sites in Fe_1_-2D-NOC; ORR reaction pathway of various possible active sites in Fe_1_-2D-NOC; simulated charge distribution in various possible active sites in Fe_1_-2D-NOC; comparison of the electron density around Fe-N_4_ and FeO-N_3_O active sites; and the effects when different Dicy:urea ratios were used (PDF)
