# Supplementary material for: General Pyrolysis for High-Loading Transition Metal Single Atoms on 2D-Nitro-Oxygeneous Carbon as Efficient ORR Electrocatalysts
Source: ACS Appl Mater Interfaces. 2024 Feb 17;16(8):10227–37. doi: 10.1021/acsami.3c18548 (PMC10910467; doi:10.1021/acsami.3c18548)
Supplement: Supplementary file 1 — am3c18548_si_001.pdf [file am3c18548_si_001.pdf]

Supporting Information

General Pyrolysis for High-loading Transition-metal Single Atoms on 2D-nitro-oxygeneous Carbon as Efficient ORR Electrocatalysts

Teera Butburee[a, b†]*, *Jitprabhat Ponchai*[a†], *Pongtanawat Khemthong*[a], *Poobodin Mano*[a], *Pongkarn Chakthranont*[a], *Saran Youngjan*[a], *Jakkapop Phanthasri*[a], *Supawadee Namuangruk*[a], *Kajornsak Faungnawakij*[a], *Xingya Wang*[b], *Yu Chen*[b], *Lijuan Zhang*[b]*

[a]National Nanotechnology Center, National Science and Technology Development Agency, 111 Thailand Science Park, Pathum Thani 12120, Thailand

[b]Shanghai Synchrotron Radiation Facility, Shanghai Advanced Research Institute, Chinese Academy of Sciences (CAS), No 239, Zhangheng Rd., New Pudong District, Shanghai 201204, P.R. China

[†]These authors contribute to this work equally.

E-mail: teera.but@nanotec.or.th; zhanglijuan@sari.ac.cn

Contents

S1: EDS Mappings of various M_1-2D-NOCs. 2

S2: High-resolution HAADF STEM images showing the metal population in the sample 4

S3: XRD of various M_1-2D-NOCs 6

S4: Rotating speed-dependent LSV of various catalysts 7

S5: ORR performance of different types of metal single atoms supported on N-doped carbon. 8

S6: Stability and methanol tolerance tests 9

S7: BET surface area and pore size distribution 10

S8: EXAFS fitting parameters 10

S9: XANES, and 1st derivative of the other M_1-2D-NOCs 11

S10: Simulated structures of possible active sites in Fe_1-2D-NOC 12

S11: ORR reaction pathway of various possible active sites in Fe_1-2D-NOC 14

S12: simulated charge distribution in various possible active sites in Fe_1-2D-NOC 15

S13: Comparison of the electron density around $Fe\text{-}N_4$ and $FeO\text{-}N_3O$ active sites 16

S14: the effects when different Dicy:Urea ratios were used 17

References 17

S1: EDS Mappings of various M₁-2D-NOCs.

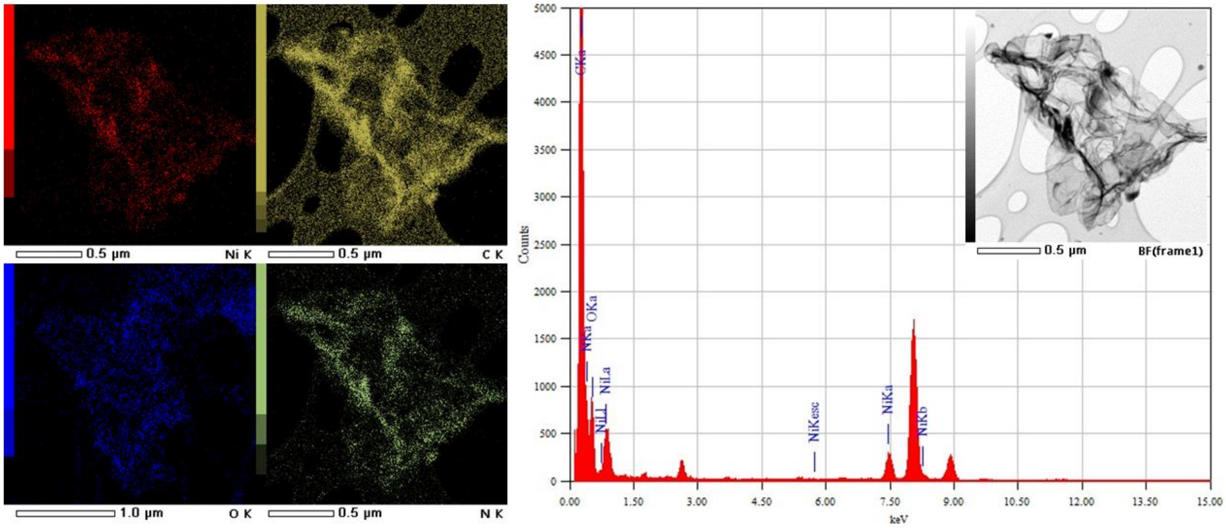

(a)

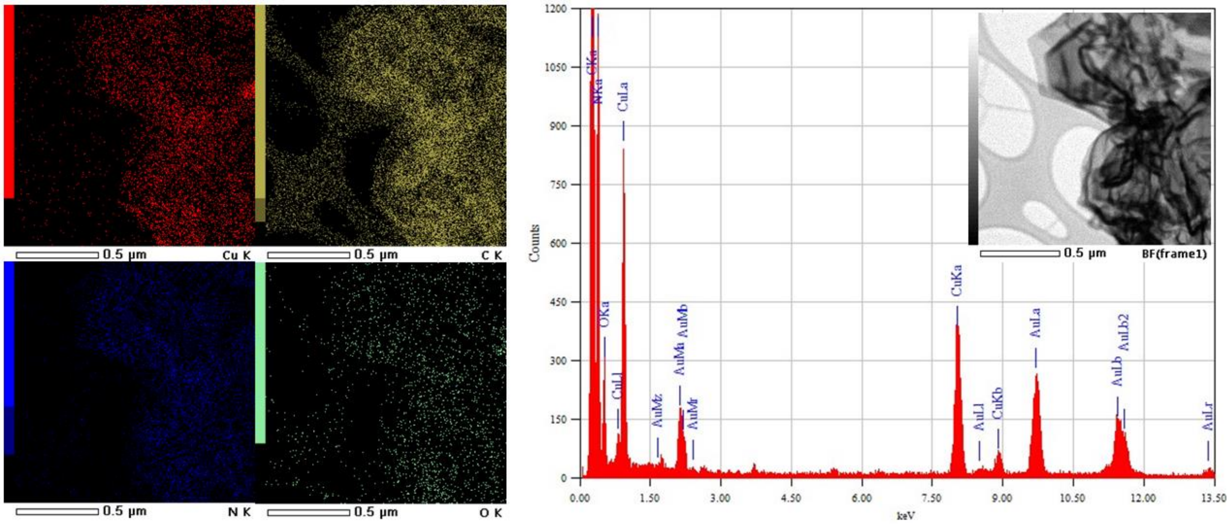

(b)

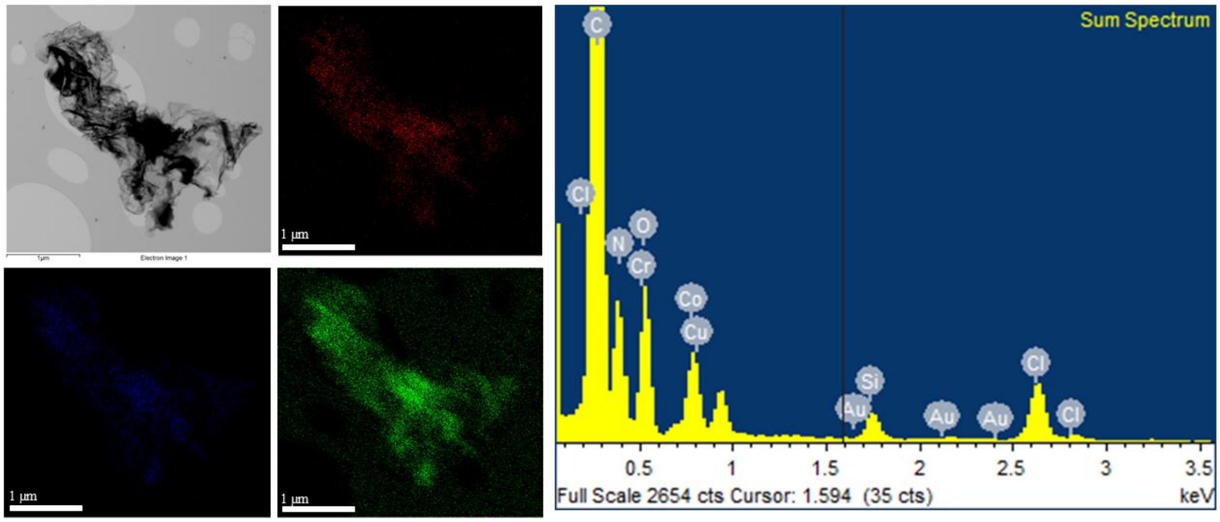

(c)

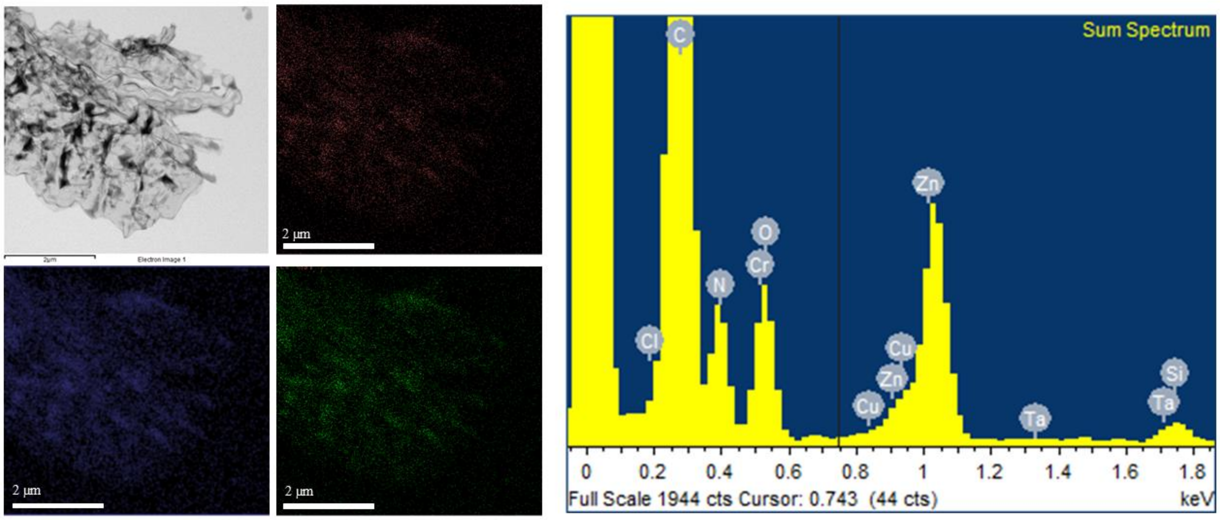

(d)

Figure S1 EDS mapping and spectrum of Ni_1-2D-NOC (a), Cu_1-2D-NOC (b), Co_1-2D-NOC (c) and Zn_1-2D-NOC (d), respectively.

S2: High-resolution HAADF STEM images showing the metal population in the sample

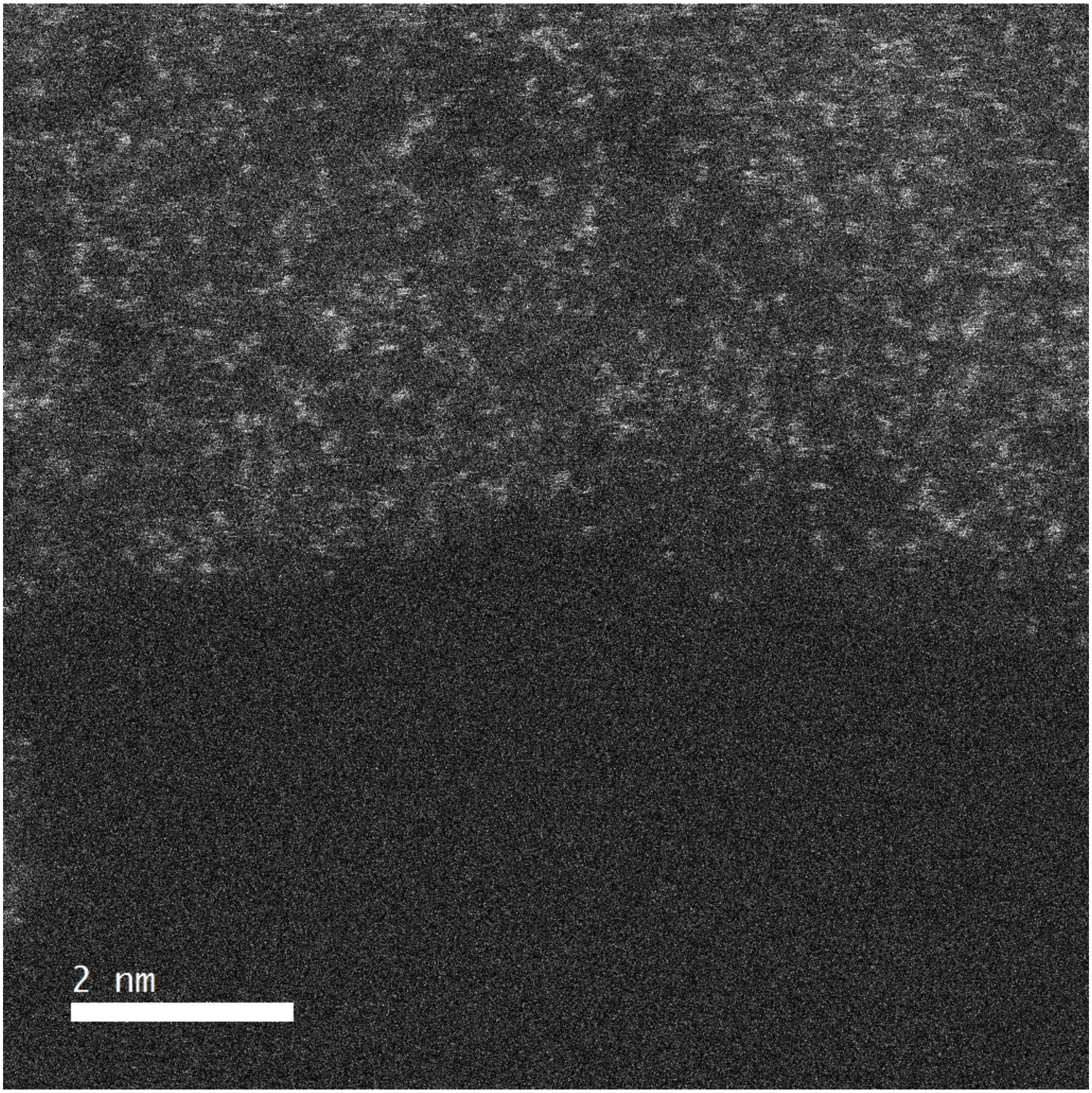

(a)

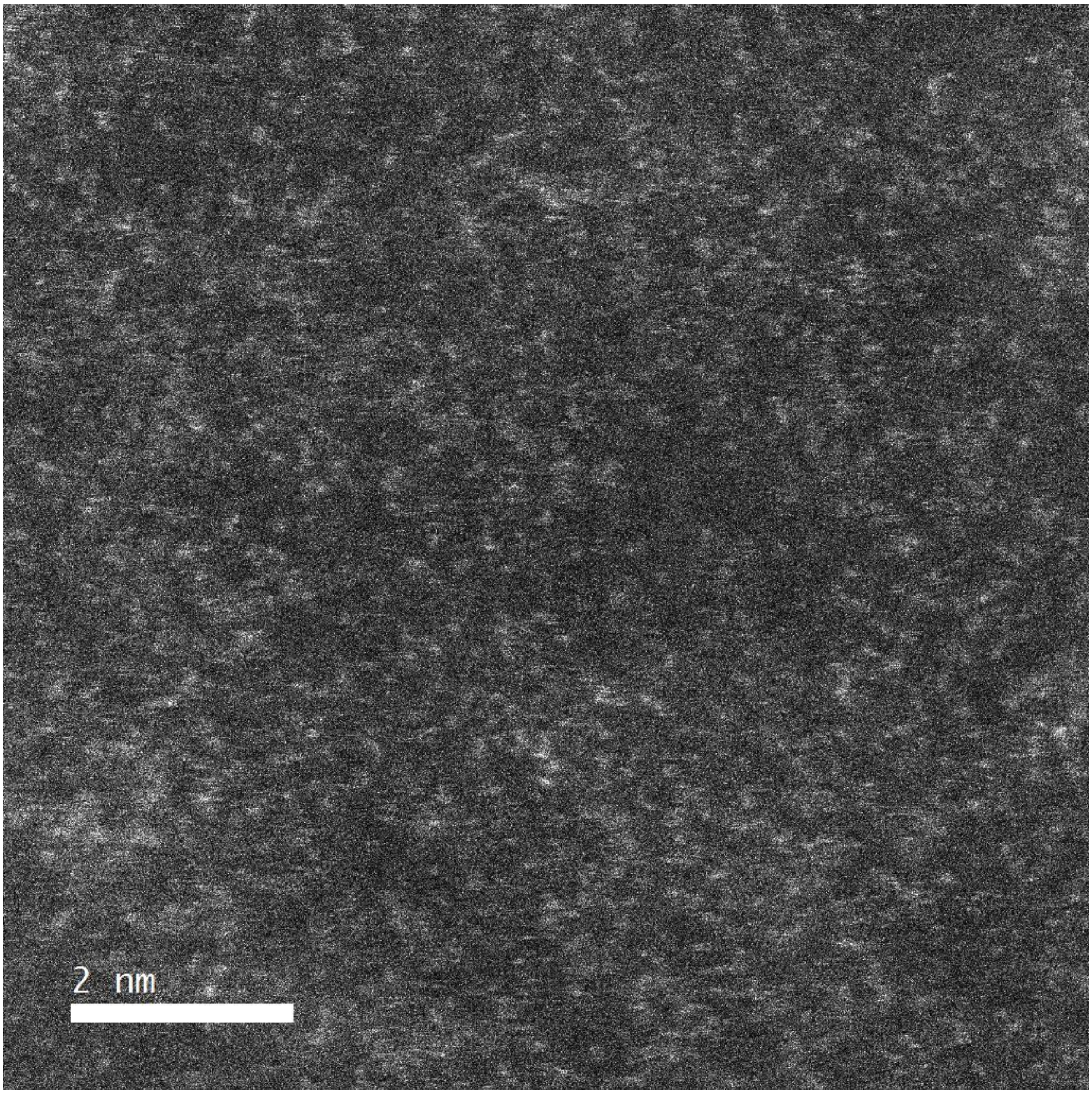

(b)

Figure S2 High-resolution HAADF STEM images showing high-density of the metal single atoms on 2D-NOC. Bright spots represent the metal atoms, while the dark media represents the 2D-NOC support, which is constructed by the lighter-weight elements including N, O, and C. **a)** the HAADF STEM images taken at the edge of the 2D nanosheet, showing the clear contrast between the metal-containing area on M_1-2D-NOC (bright area), and the background (the dark area without bright spots). **b)** the HAADF STEM images taken at the middle of the M_1-2D-NOC nanosheet, showing high density and uniform distribution of the isolated metal atoms throughout the nanosheet.

S3: XRD of various M_1-2D-NOCs

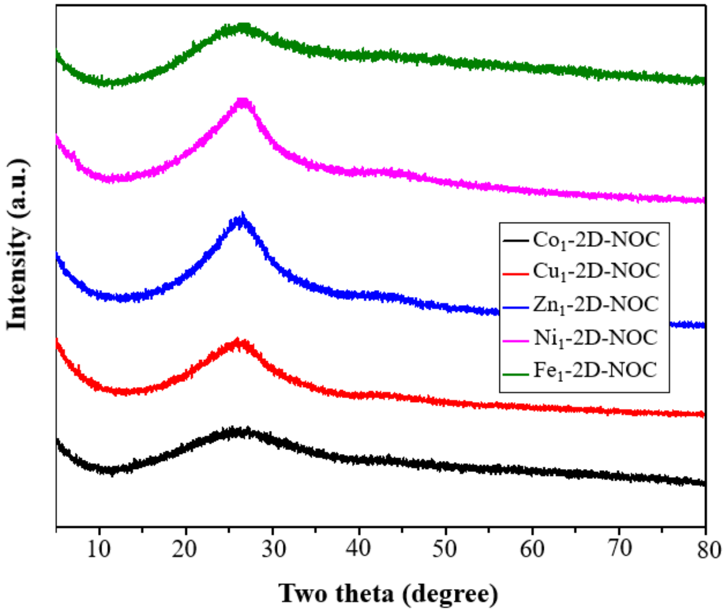

Figure S3 XRD patterns of various M_1-2D-NOCs

S4: Rotating speed-dependent LSV of various catalysts

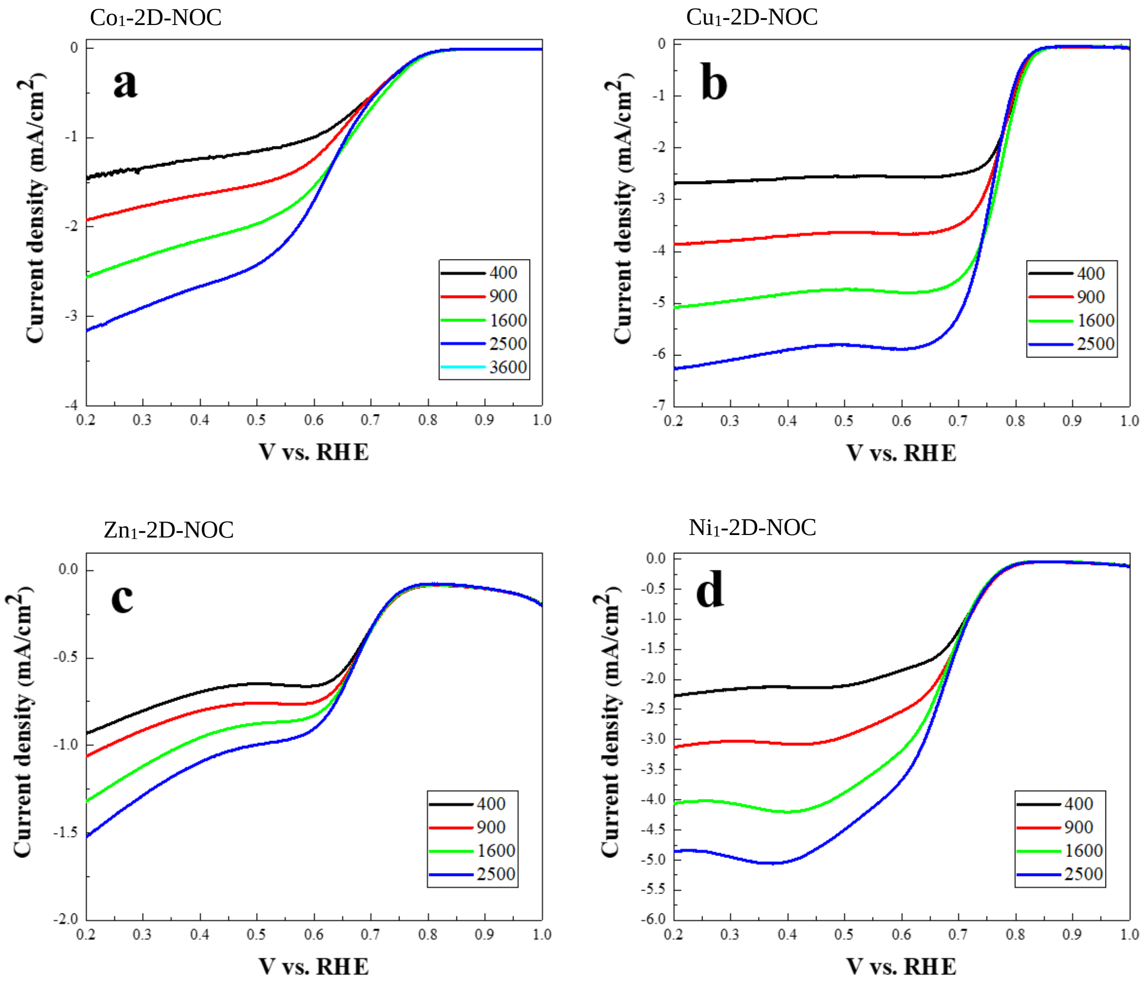

Figure S4 a-d) LSV curves at variable speeds of Co_1-2D-NOC, Cu_1-2D-NOC, Zn_1-2D-NOC, and Ni_1-2D-NOC, respectively.

S5: ORR performance of different types of metal single atoms supported on N-doped carbon.

Active site / Support	Onset potential (V)	Half wave potential (V)	Tafel slope (mV/dec)	limiting J @1600rpm (mA/cm^2)	References
**Fe_1-2D-NOC**	**0.985**	**0.826**	**40.86**	**5.22**	**This work**
$Fe-N_4$/N-doped porous carbon from Molten salt	0.95	0.84	46	4.67	Nano Energy, **2020**, 72, 104670.[1]
Zn/nitrogen doped carbon	0.98	0.873	-	5.02	Angew. Chem. Int. Ed. **2019**, 58, 7035–7039.[2]
arm. N_4O_2-Cu_1^2/N-doped carbon	0.92	0.81	53.77	5.31	Small. **2022,** 2204767.[3]
$Cu-N_x$/S, N-codoped nanocarbon	0.96	0.84	56	5.05	Nano Res. **2022**, 15, 5995-6000.[4]
Cu/macro-porous N-doped carbon	0.95	0.87	78	5.40	Chinese Chem. Lett. **2022**, 33, 2585-2589.[5]
ZnCo/N-doped carbon	1.004	0.861	67	6.10	Angew. Chem. Int. Ed., **2019**, 58(9), 2622–2626.[6]
$Zn-N_2$/N-doped carbon	0.95	0.857	27	5.90	Nat. Commun., **2019**, 10, 1, 2623.[7]
Fe/N-doped carbon	0.950	0.892	89.2	5.27	Energy Storage Mater., **2021**, 35, 723–730.[8]
Ni/N-doped carbon	0.942	0.846	103.1	5.35	
FeNi/N-doped carbon	0.952	0.907	74.6	5.51	
$Fe-N_4$/hydrangea-like carbon from ZIF-8	0.988	0.858	56	6.68	Chem. Eng. J., **2022**, 429, 132307.[9]
Co-N-C/doped carbon from acetate/MOF	1.01	0.89	59	6.10	Small. **2021**, 17, 2104684.[10]
Co/N-doped carbon flake on carbon nanofibers from ZIF/ENF	1.05	0.88	56	5.91	Adv. Mater. **2019**, 31, 1808267.[11]
Ni/N-doped graphene nanosheets	0.885	0.767	68	4.54	Nat. Commun., **2021**, 12, 1, 5589.[12]
Ni-NC/N-doped carbon	0.945	0.766	133	5.19	Small, **2023**, 19(8), 2206071.[13]
$Ni-Si_1N_3$/N-doped carbon	0.951	0.866	75	5.78	
Fe-Nx/N-doped graphene	1.01	0.87	-	6.82	ACS Cent. Sci. **2020**, 6, 1431-1440.[14]
$Co-N_x$/N-doped graphene	0.92	0.84	-	5.46	
$Cu-N_x$/N-doped graphene	0.90	0.79	-	5.57	
$Ni-N_x$/N-doped graphene	0.84	0.76	-	4.53	
$Fe-N_4$/ N-doped porous carbon	0.986	0.90	58	6.10	Angew. Chem. Int. Ed. **2017**, 56, 6937–6941.[15]
Fe-Nx /N doped porous carbon	1.0	0.93	57.2	6.0	Adv. Mater. **2020**, 32, 1907399.[16]

S6: Stability and methanol tolerance tests

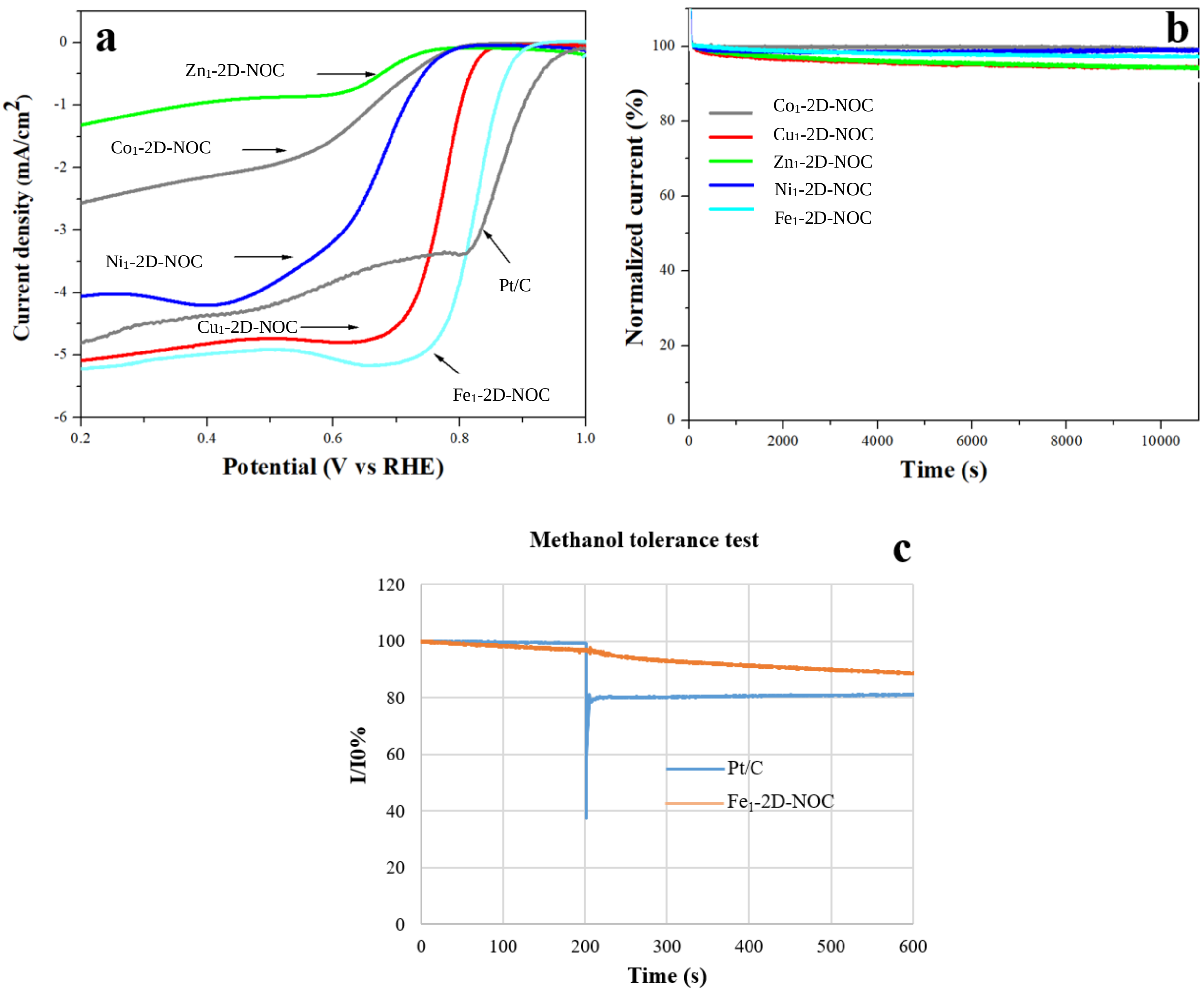

Figure S6 a) ORR polarization plots of various M_1-2D-NOCs, compared to that of the commercial Pt/C. **b)** Chronoamperometric responses (normalized to the starting currents) of various M_1-2D-NOCs at a rotating speed of 1600 rpm, the potential was controlled at 0.3 V (vs. RHE) in O_2-saturated 0.1 M KOH solution for 3 h. **c)** Chronoamperometric responses with and without methanol to compare methanol tolerance between Fe_1-2D-MOC and the commercial Pt/C. Methanol was introduced to the system at Time = 200s.

As shown in Figure S6 a, basically, Fe_1-2D-NOC still has slightly inferior onset (E_{onset}) and half-wave ($E_{1/2}$) potentials those of the benchmark Pt/C catalyst, but it has much better stability (Figure S6 b) and methanol tolerance (Figure S6 c) in our tests. However, please note that the performance of the commercial Pt/C seems also dependent on the producing company, as the commercial Pt/C we purchased (Tanaka Kikinzoku International Co. Ltd.) shows significantly higher E_{onset} and $E_{1/2}$ than those reported in the literature.[17-19] As shown in Figure S6 c, the current of Pt/C is sharply dropped after adding methanol (at 200 s), while that of Fe_1-2D-NOC slightly decay. This evidence suggests that Fe_1-2D-NOC has much higher methanol tolerance.

S7: BET surface area and pore size distribution

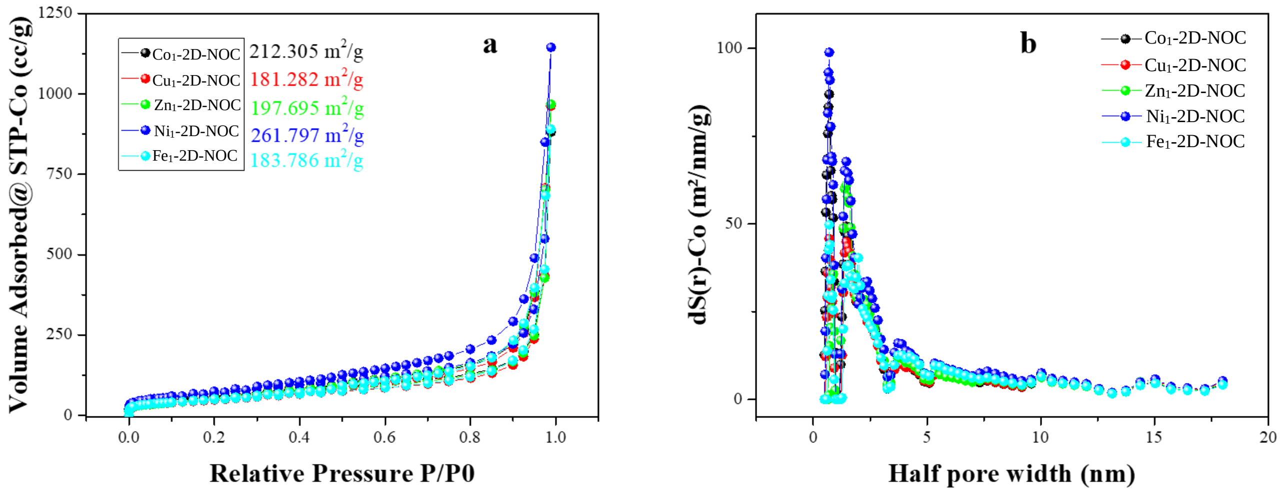

Fig S7 a) N_2 adsorption-desorption isotherms. **b)** pore size distribution of various M_1-2D-NOCs**.**

S8: EXAFS fitting parameters

Table S8: EXAFS fitting parameters derived from fitting Fe_1-2D-NOC

Path name	CN	σ^2	R	E_0
Fe-O	1.95	0.0040	1.96	4.74
Fe-N	2.92	0.0046	2.12	

where S_0^2 is 0.974

EXAFS fitting parameters. $\sigma^2 < 0.1$ indicates high reliability of the fitting results.

S9: XANES, and 1st derivative of the other M_1-2D-NOCs

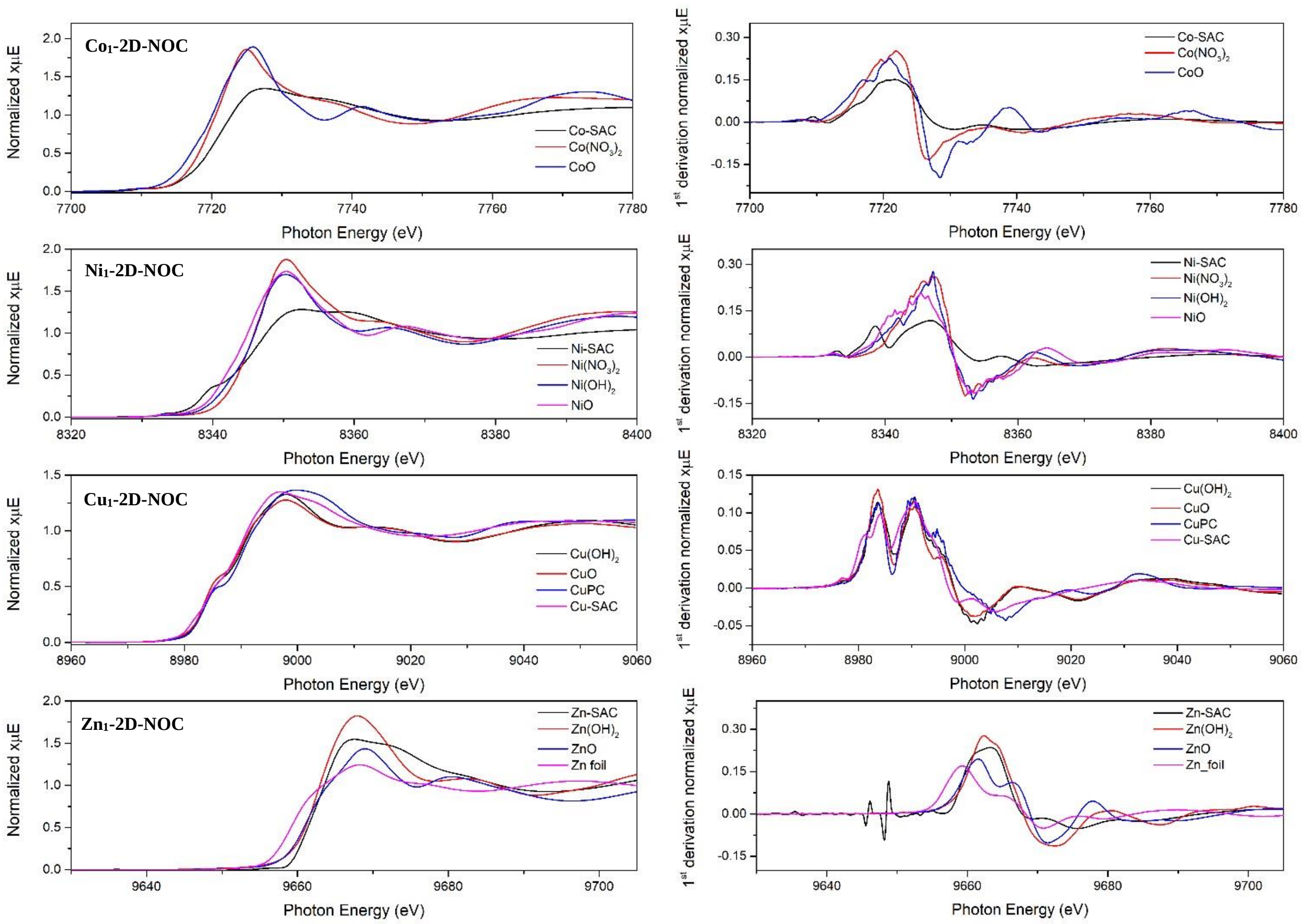

Figure S9 XANES spectra of the other M_1-2D-NOCs compared to that of various standard materials (left column), and their 1st shell derivative.

S10: Simulated structures of possible active sites in Fe1-2D-NOC

Table S10 The model structures used in this study to analysis the synthesized material.

(1) Fe-N_4<br>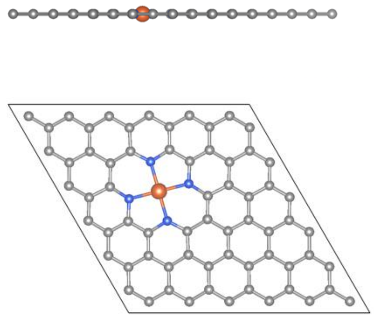	(2) Fe-N_3O<br>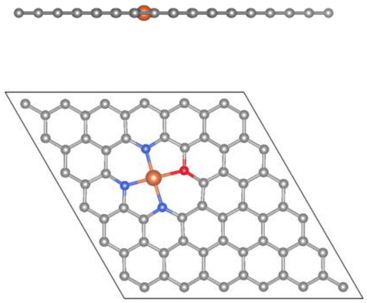	(3) Fe-N_2O_2<br>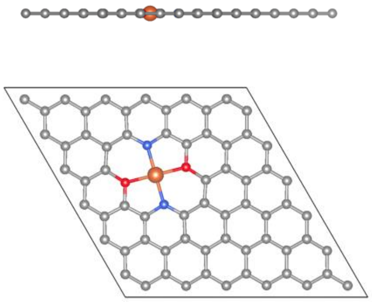
(4) FeO-N_4<br>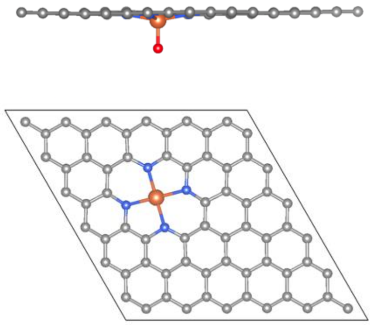	(5) FeO-N_3O (most possible)<br>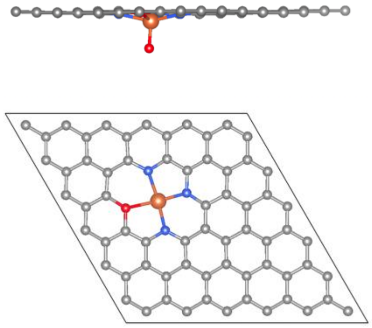	(6) armchair Fe_2-N_6<br>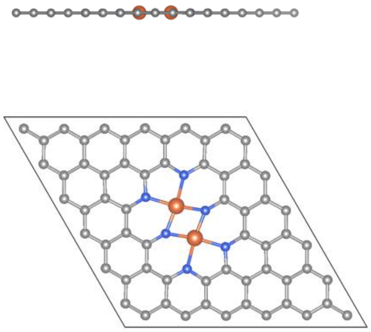
(7) armchair Fe_2-N_5O<br>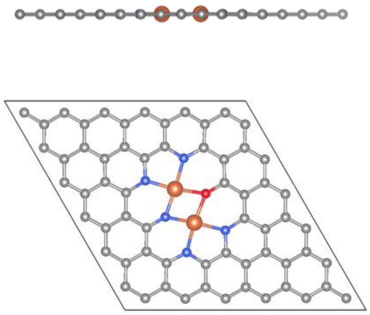	(8) armchair Fe_2-N_4O_2<br>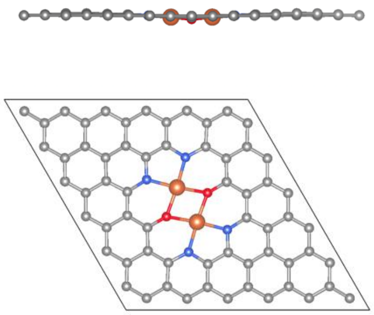	(9) zigzag Fe_2-N_6<br>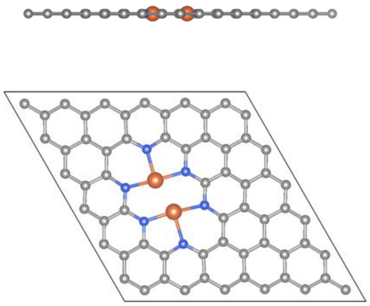
(10) zigzag Fe_2-N_5O<br>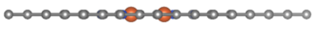	(11) zigzag Fe_2-N_4O_2<br>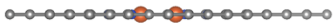	(12) Fe_1-N_5<br>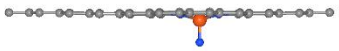

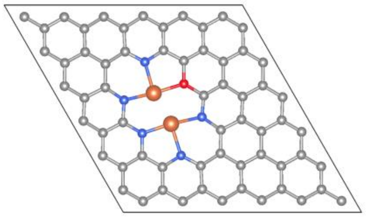	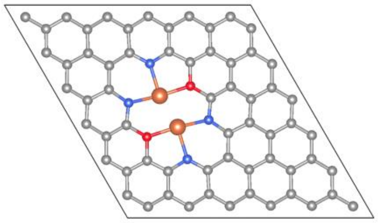	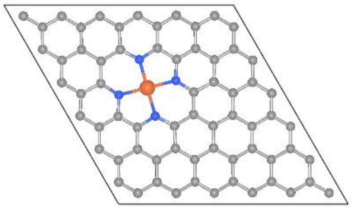

S11: ORR reaction pathway of various possible active sites in Fe_1-2D-NOC

(1) $Fe-N_4$

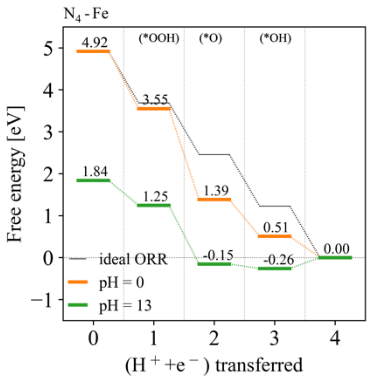

(2) $Fe-N_3O$

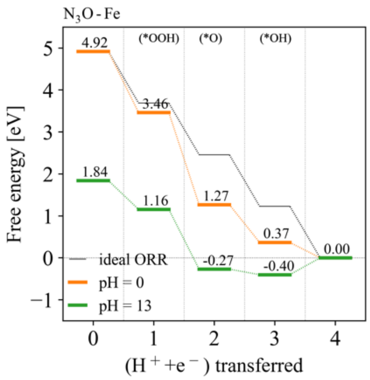

(3) $Fe-N_2O_2$

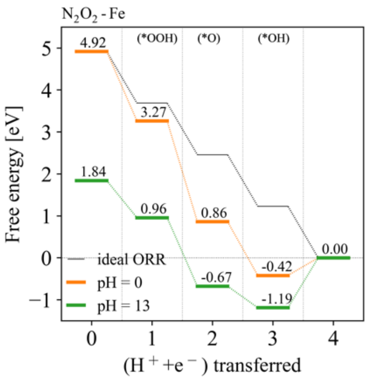

(4) $FeO-N_4$

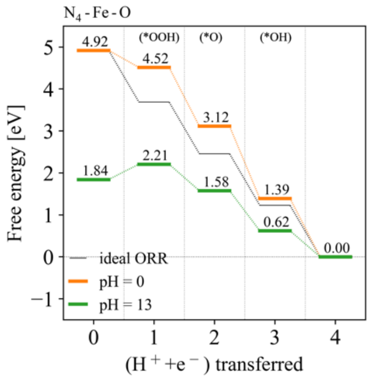

(5) $FeO-N_3O$

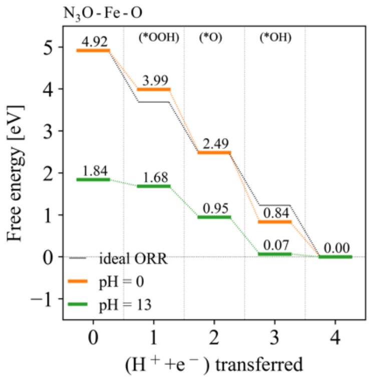

(6) armchair Fe_2-N_6

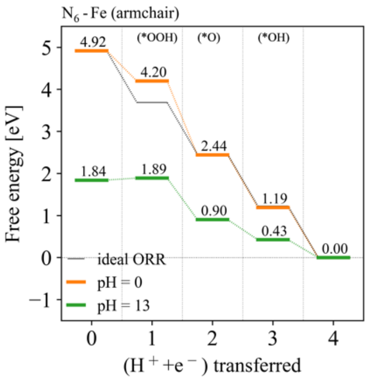

(7) armchair Fe_2-N_5O

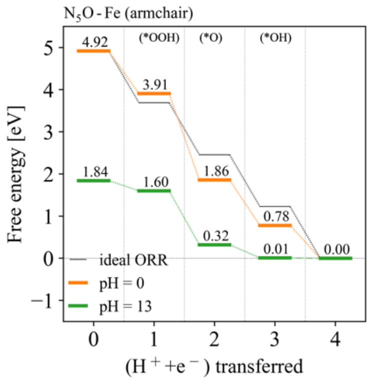

(8) armchair $Fe_2-N_4O_2$

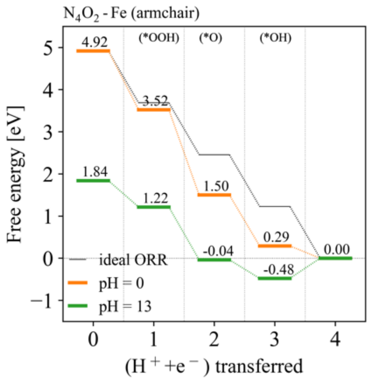

(9)

Figure S11 The ORR free energy pathways of 8 model structures at pH = 0 (orange) and 13 (green).

S12: simulated charge distribution in various possible active sites in Fe_1-2D-NOC

Table S12 The visualization of the Bader charge distribution on each atom in each structure, when blue represents positive charge and red represents negative charge.

<table>
<tr>
<td>(1) $Fe-N_4$
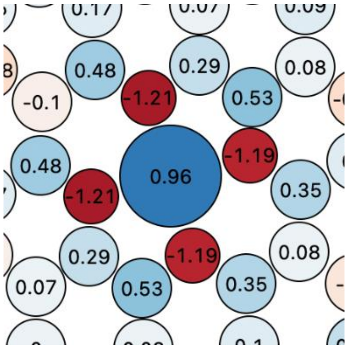
</td>
<td>(2) $Fe-N_3O$
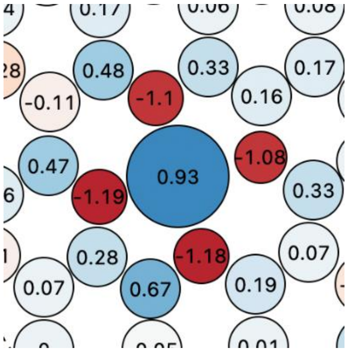
</td>
<td>(3) $Fe-N_2O_2$
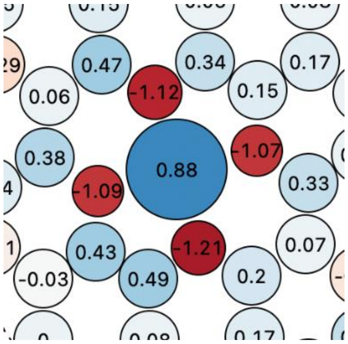
</td>
</tr>
<tr>
<td>(4) $FeO-N_4$
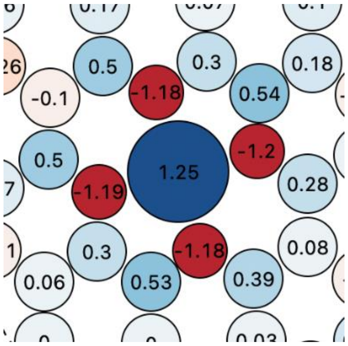
</td>
<td>(5) $FeO-N_3O$
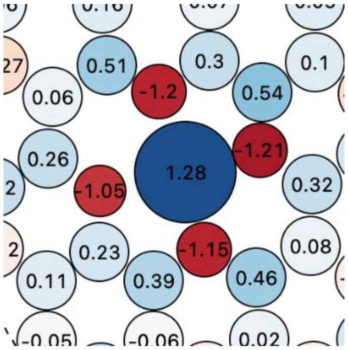
</td>
<td>(6) armchair Fe_2-N_6
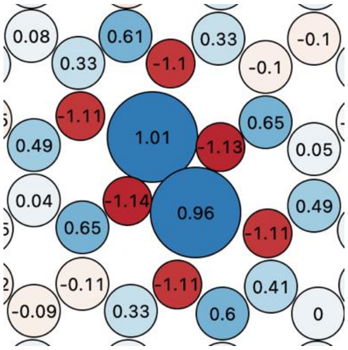
</td>
</tr>
<tr>
<td>(7) armchair Fe_2-N_5O
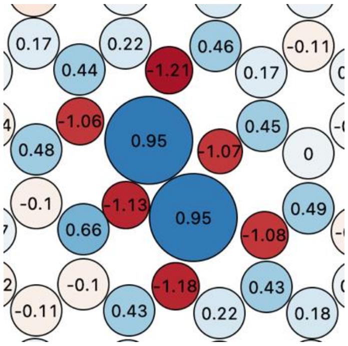
</td>
<td>(8) armchair $Fe_2-N_4O_2$
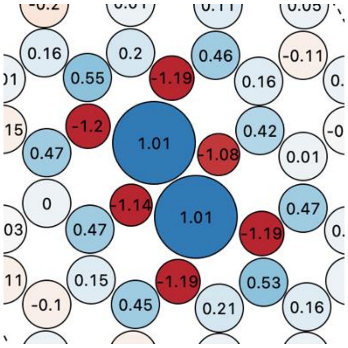
</td>
<td>(9) zigzag Fe_2-N_6
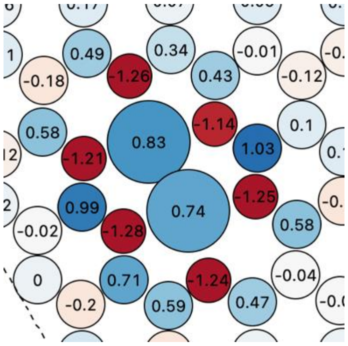
</td>
</tr>
<tr>
<td>(10) zigzag Fe_2-N_5O</td>
<td>(11) zigzag $Fe_2-N_4O_2$</td>
<td></td>
</tr>
</table>

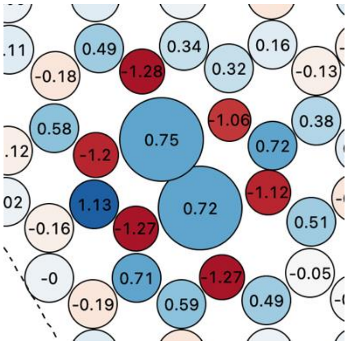	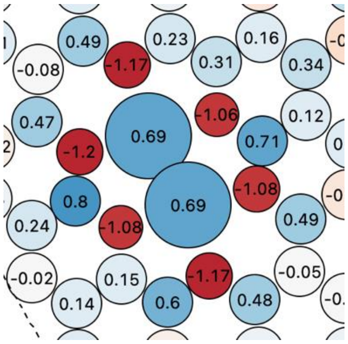	

S13: Comparison of the electron density around Fe-N_4 and FeO-N_3O active sites.

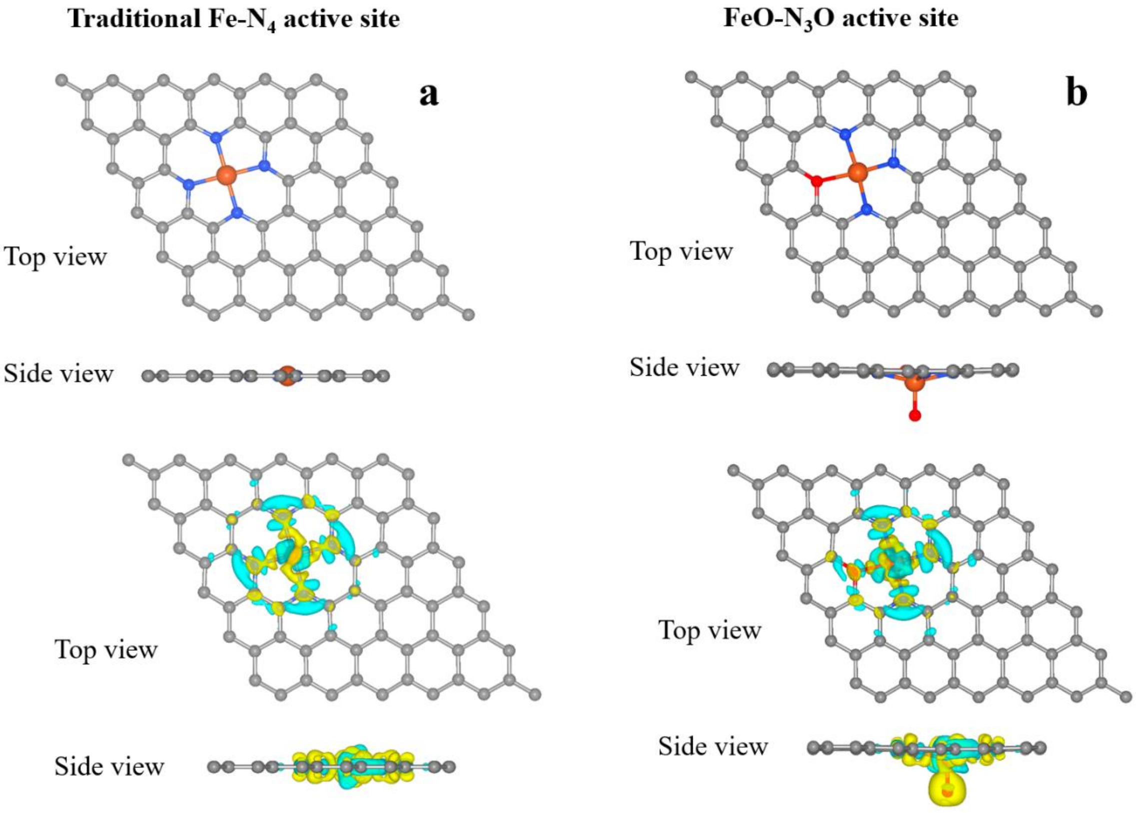

Figure 13 a) electron density distribution around the traditional Fe-N_4 active site compared to **b)** electron density distribution around the FeO-N_3O active site. (yellow is the electron cloud with higher e^- density, cyan is the electron cloud with lower e^- density)

S14: the effects when different Dicy:Urea ratios were used.

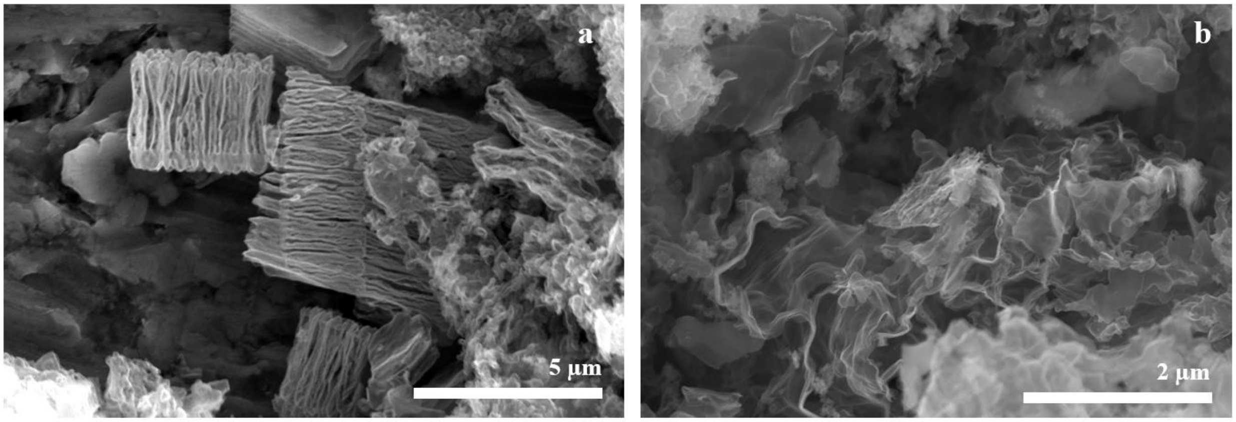

Figure S14 a) the structure of the resultant product when M-Phc:Urea:Dicy of 0.5:10:0 g was used. b) the structure of the resultant product when M-Phc:Urea:Dicy of 0.5:5:5 g was used (M= Cu in this case study).

References

1. Hu, J.; Wu, D.; Zhu, C.; Hao, C.; Xin, C.; Zhang, J.; Guo, J.; Li, N.; Zhang, G.; Shi, Y., Melt-salt-assisted Direct Transformation of Solid Oxide Into Atomically Dispersed FeN_4 Sites on Nitrogen-doped Porous Carbon. *Nano Energy* 2020, *72*, 104670.
2. Li, J.; Chen, S.; Yang, N.; Deng, M.; Ibraheem, S.; Deng, J.; Li, J.; Li, L.; Wei, Z., Ultrahigh-Loading Zinc Single-atom Catalyst for Highly Efficient Oxygen Reduction in Both Acidic and Alkaline Media. *Angew. Chem. Int. Ed.* 2019, *58* (21), 7035-7039.
3. Butburee, T.; Ponchai, J.; Meeporn, K.; Phawa, C.; Chakthranont, P.; Khemthong, P.; Mano, P.; Namuangruk, S.; Chinsirikul, W.; Faungnawakij, K., New Folding 2D-Layered Nitro-Oxygenated Carbon Containing Ultra High-Loading Copper Single Atoms. *Small* 2022, 2204767.
4. Zhang, H.; Sun, Q.; He, Q.; Zhang, Y.; He, X.; Gan, T.; Ji, H., Single Cu atom Dispersed on S, N-codoped Nanocarbon Derived from Shrimp Shells for Highly-efficient Oxygen Reduction Reaction. *Nano Res.* 2022, *15* (7), 5995-6000.
5. Ma, J.; Liu, B.; Wang, R.; Sun, Z.; Zhang, Y.; Sun, Y.; Cai, Z.; Li, Y.; Zou, J., Single-Cu-atoms Anchored on 3D Macro-porous Carbon Matrix as Efficient Catalyst for Oxygen Reduction and Pt Co-catalyst for Methanol Oxidation. *Chin. Chem. Lett.* 2022, *33* (5), 2585-2589.
6. Lu, Z.; Wang, B.; Hu, Y.; Liu, W.; Zhao, Y.; Yang, R.; Li, Z.; Luo, J.; Chi, B.; Jiang, Z., An Isolated Zinc–cobalt Atomic Pair for Highly Active and Durable Oxygen Reduction. *Angew. Chem. Int. Ed.* 2019, *131* (9), 2648-2652.
7. Li, F.; Bu, Y.; Han, G.-F.; Noh, H.-J.; Kim, S.-J.; Ahmad, I.; Lu, Y.; Zhang, P.; Jeong, H. Y.; Fu, Z., Identifying the Structure of Zn-N_2 Active Sites and Structural Activation. *Nat. Commun.* 2019, *10* (1), 2623.
8. Luo, F.; Zhu, J.; Ma, S.; Li, M.; Xu, R.; Zhang, Q.; Yang, Z.; Qu, K.; Cai, W.; Chen, Z., Regulated Coordination Environment of Ni Single Atom Catalyst Toward High-efficiency Oxygen Electrocatalysis for Rechargeable Zinc-air Batteries. *Energy Storage Mater.* 2021, *35*, 723-730.

9. Shao, C.; Wu, L.; Wang, Y.; Qu, K.; Chu, H.; Sun, L.; Ye, J.; Li, B.; Wang, X., An Open Superstructure of Hydrangea-like Carbon with Highly Accessible Fe-N_4 Active Sites for Enhanced Oxygen Reduction Reaction. *Chem. Eng. J.* 2022, *429*, 132307.
10. Gao, J.; Hu, Y.; Wang, Y.; Lin, X.; Hu, K.; Lin, X.; Xie, G.; Liu, X.; Reddy, K. M.; Yuan, Q., MOF Structure Engineering to Synthesize Co-N-C Catalyst with Richer Accessible Active Sites for Enhanced Oxygen Reduction. *Small* 2021, *17* (49), 2104684.
11. Ji, D.; Fan, L.; Li, L.; Peng, S.; Yu, D.; Song, J.; Ramakrishna, S.; Guo, S., Atomically Transition Metals on Self-supported Porous Carbon Flake Arrays as Binder-free Air Cathode for Wearable zinc−air Batteries. *Adv. Mater.* 2019, *31* (16), 1808267.
12. Wan, W.; Zhao, Y.; Wei, S.; Triana, C. A.; Li, J.; Arcifa, A.; Allen, C. S.; Cao, R.; Patzke, G. R., Mechanistic Insight into the Active Centers of Single/dual-atom Ni/Fe-based Oxygen Electrocatalysts. *Nat. Commun.* 2021, *12* (1), 5589.
13. Wang, F.; Li, Y.; Zhang, R.; Liu, H.; Zhang, Y.; Zheng, X.; Zhang, J.; Chen, C.; Zheng, S.; Xin, H. L., Activating Single-Atom Ni Site via First-Shell Si Modulation Boosts Oxygen Reduction Reaction. *Small* 2023, *19* (8), 2206071.
14. Meng, J.; Li, J.; Liu, J.; Zhang, X.; Jiang, G.; Ma, L.; Hu, Z.-Y.; Xi, S.; Zhao, Y.; Yan, M., Universal Approach to Fabricating Graphene-supported Single-atom Catalysts from Doped ZnO Solid Solutions. *ACS Cent. Sci.* 2020, *6* (8), 1431-1440.
15. Chen, Y.; Ji, S.; Wang, Y.; Dong, J.; Chen, W.; Li, Z.; Shen, R.; Zheng, L.; Zhuang, Z.; Wang, D., Isolated Single Iron Atoms Anchored on N-doped Porous Carbon as an Efficient Electrocatalyst for the Oxygen Reduction Reaction. *Angew. Chem. Int. Ed.* 2017, *56* (24), 6937-6941.
16. Chen, G.; Liu, P.; Liao, Z.; Sun, F.; He, Y.; Zhong, H.; Zhang, T.; Zschech, E.; Chen, M.; Wu, G., Zinc-mediated Template Synthesis of Fe-N-C Electrocatalysts with Densely Accessible Fe-N_x Active Sites for Efficient Oxygen Reduction. *Adv. Mater.* 2020, *32* (8), 1907399.
17. Han, Y.; Wang, Y.; Xu, R.; Chen, W.; Zheng, L.; Han, A.; Zhu, Y.; Zhang, J.; Zhang, H.; Luo, J., Electronic Structure Engineering to Boost Oxygen Reduction Activity by Controlling the Coordination of the Central Metal. *Energy Environ. Sci.* 2018, *11* (9), 2348-2352.
18. Yang, Z.; Chen, B.; Chen, W.; Qu, Y.; Zhou, F.; Zhao, C.; Xu, Q.; Zhang, Q.; Duan, X.; Wu, Y., Directly Transforming Copper (I) Oxide Bulk Into Isolated Single-atom Copper Sites Catalyst Through Gas-transport Approach. *Nat. Commun.* 2019, *10* (1), 1-7.
19. Jiang, R.; Li, L.; Sheng, T.; Hu, G.; Chen, Y.; Wang, L., Edge-site Engineering of Atomically Dispersed Fe–N_4 by Selective C–N Bond Cleavage for Enhanced Oxygen Reduction Reaction Activities. *J. Am. Chem. Soc.* 2018, *140* (37), 11594-11598.
